# Multi-scale resistivity imaging for soil moisture and structure characterization in precision agriculture

**DOI:** 10.1371/journal.pone.0350034

**Published:** 2026-06-16

**Authors:** Widodo, Abdul Salam, Fatkhan, Tedy Setiawan

**Affiliations:** 1 Applied Geophysics and Exploration Expertise Group, Geophysical Engineering, Faculty of Mining and Petroleum Engineering, Bandung Institute of Technology, Bandung, Indonesia; 2 Seismology Exploration and Engineering Expertise Group, Geophysical Engineering, Faculty of Mining and Petroleum Engineering, Bandung Institute of Technology, Bandung, Indonesia; National Research and Innovation Agency, INDONESIA

## Abstract

Sustainable agriculture in tropical regions relies on precise understanding of soil water dynamics under varying topographic, textural, and climatic conditions. This study integrates Electrical Resistivity Tomography (ERT) and Electromagnetic Induction (EMI) to characterize soil moisture distribution and subsurface textural heterogeneity across three contrasting agricultural landscapes in West Java, Indonesia—Subang (coastal lowlands), Bandung (uplands), and Sumedang (terraced highlands). ERT provided high-resolution vertical profiles to 5 m depth, revealing resistivity ranges that correspond to lithological and hydrological properties. In Subang, low resistivity (1.7–30 Ω·m) showed high-salinity clay loam with a shallow water table. Conversely, high resistivity in Bandung (70–300 Ω·m) reflected well-drained sandy layers with limited retention. Intermediate values in Sumedang suggested deep moisture storage within clay-rich layers beneath drier topsoil, influenced by terrace morphology. EMI mapping complemented ERT by capturing lateral resistivity variations at fixed depths, offering spatial continuity across the surveyed areas. The combined approach revealed that slope gradient, soil texture, and drainage conditions jointly govern water retention and availability. The integration of ERT and EMI provides complementary information on vertical and lateral variability of soil moisture distribution. Field observations and soil profile analysis confirm the reliability of the geophysical interpretation. These findings demonstrate that integrated geophysical imaging provides an effective non-invasive tool for mapping soil moisture variability and supporting precision agriculture strategies in tropical agricultural environments.

## 1. Introduction

Monitoring soil water content (SWC) is a fundamental pillar of sustainable agricultural management in tropical regions, where extreme rainfall variability directly affects crop productivity [1–3]. Precise soil moisture data are vital for improving crop yields, enhancing irrigation water efficiency, and mitigating risks of land degradation such as soil compaction and erosion [4,5]. In tropical zones facing climate change challenges, accurate SWC monitoring serves as a basis for adaptation strategies to support food security and the sustainability of agricultural ecosystems [6–8].

Furthermore, stable soil water availability is a determining factor in plant metabolic processes and the efficiency of nutrient uptake from the soil profile to the roots [9–11]. High-resolution and continuous moisture measurements enable the implementation of precision agriculture, where irrigation and fertilization interventions can be conducted in a prompt manner to prevent resource waste and environmental pollution due to nutrient leaching [12–14]. Advanced monitoring technologies are now an urgent requirement to understand subsurface water dynamics across various soil textures and complex topographies in tropical landscapes [15–17].

Despite its critical importance, obtaining high-resolution spatial moisture data is still a significant logistical and technical challenge. Conventional methods, such as frequent gravimetric sampling and laboratory analyses, are often prohibitively expensive, labor-intensive, and impractical for large-scale or real-time applications [3,18,19]. These traditional approaches are fundamentally time-consuming and provide data only at discrete points, not capturing the high spatial heterogeneity inherent in soil moisture and texture [3,19]. Consequently, there is a clear scientific need for non-invasive technologies that can rapidly, reliably, and affordably provide comprehensive soil moisture data at the field scale [20–22].

Near-surface geophysical methods offer a promising, non-invasive alternative for characterizing subsurface conditions. Techniques such as Electrical Resistivity Tomography (ERT), Electromagnetic Induction (EMI), and Ground-Penetrating radar (GPR) have been successfully applied to map soil moisture and related properties globally [23–25]. ERT measures subsurface resistivity distributions by injecting current, where lower resistivity typically shows higher moisture or clay content [16,26–28]. Integrating these geophysical sensors has been proven to significantly improve soil water content estimation accuracy by streamlining the identification of zones susceptible to water accumulation [23–25].

In Indonesia, the application of near-surface geophysics for precision farming is still emerging, but recent works have shown promising results. Widodo, 2024 [29] demonstrated that a combination of ERT and EMI surveys can effectively characterize soil moisture distribution in a West Java agricultural field, with resistivity values ranging from ~15–170 Ω·m and conductivity from 2 to 115 mS/m correlating well with soil moisture observed in test pits. That study confirmed a good correspondence between geophysical measurements and actual soil conditions (e.g., finding a resistive, drier layer at ~0.75 m depth above a conductive, wetter zone at ~1.5 m) and highlighted the utility of geophysics for guiding precision agriculture practices. Likewise, Widodo et al.,2025a [30] applied ERT and EMI in a mountainous farming area (Ciumbuleuit, West Bandung) and found that the steep topography led to very dry topsoil overlying wetter subsoils: ERT profiles showed high resistivity values (on the order of 10^2 Ω·m) in the near-surface, with much lower resistivities (down to <60 Ω·m) at depths beyond ~0.3 m, indicating moisture retained in the deeper soil. The shallow EMI maps from that site similarly detected low conductivity (dry) conditions at the surface and higher conductivity (wet soil) at depth, suggesting that rainwater rapidly percolates downslope in the steep terrain, leaving surface soil desiccated [29]. In contrast, in a terraced highland area of Sumedang, Widodo et al.,2025b [31] reported that significant moisture was present at depth even though the surface appeared dry: low resistivity values (~8–50 Ω·m) were observed in clay-rich subsoil layers below ~0.75 m, whereas the upper layers had higher resistivity (often >100 Ω·m) due to lower moisture. This finding suggests that the terraced morphology and soil texture in Sumedang allow water to accumulate in the subsoil (which can enhance fertility and potential irrigation water reserves), even if the topsoil dries out between rains.

Despite the increasing use of near-surface geophysical methods for soil moisture investigations, significant knowledge gaps are still in understanding how different geomorphological settings influence subsurface water distribution in tropical agricultural landscapes. Most earlier studies have focused on single-site analyses or homogeneous terrains, limiting the ability to generalize results across areas with contrasting elevation, soil texture, and topographic conditions. In tropical regions such as Indonesia, where agricultural lands often occur across highly variable landscapes, there is a clear need for comparative studies that integrate geophysical measurements with environmental characteristics to better understand spatial soil moisture dynamics.

Therefore, this study aims to evaluate the capability of integrated near-surface geophysical methods, particularly Electrical Resistivity Tomography (ERT) and Electromagnetic Induction (EMI), to characterize soil water content (SWC) across three agricultural regions in West Java with contrasting geomorphological settings: Subang (lowland coastal plain), Bandung (steep upland terrain), and Sumedang (terraced highland). By combining geophysical imaging with field observations and soil characteristics, this research investigates how variations in elevation, soil texture, drainage conditions, and land morphology control subsurface moisture distribution.

The novelty of this study lies in its comparative multi-site approach in tropical agricultural systems and the integration of ERT and EMI measurements to reveal spatial moisture variability under different topographic and geological conditions. The results offer practical insights into precision agriculture and sustainable water management by proving how geophysical techniques can support rapid, non-invasive soil moisture monitoring in complex tropical environments.

## 2. Materials and methods

### 2.1. Resistivity survey (ERT)

Electrical resistivity surveying determines the subsurface’s electrical properties by injecting current into the ground through a pair of current electrodes and measuring the resulting voltage difference at a pair of potential electrodes. By systematically moving the electrodes (varying their spacing and position), we obtain measurements that can be used to infer how resistivity varies both laterally and vertically beneath the survey line. Combining vertical sounding and lateral profiling in a single process (i.e., two-dimensional resistivity imaging) enables Electrical Resistivity Tomography (ERT) to map subsurface resistivity in cross-section. The data are typically inverted to produce a model of true resistivity distribution with depth.

Resistivity, denoted ρ, is related to measured voltage and current by Ohm’s law. In resistivity surveys, the basic formula is:


$$\begin{array}{c} \rho =\text{K}\frac{\Delta \text{V}}{\text{I}} \end{array}$$ (1)

where V is the measured potential difference (volts), I is the injected current (amperes), and K is the geometric factor (meters) determined by the electrode configuration and spacing. In practice, K is calculated for a given array geometry so that the measured resistance can be converted to apparent resistivity.

**Fig 1:**
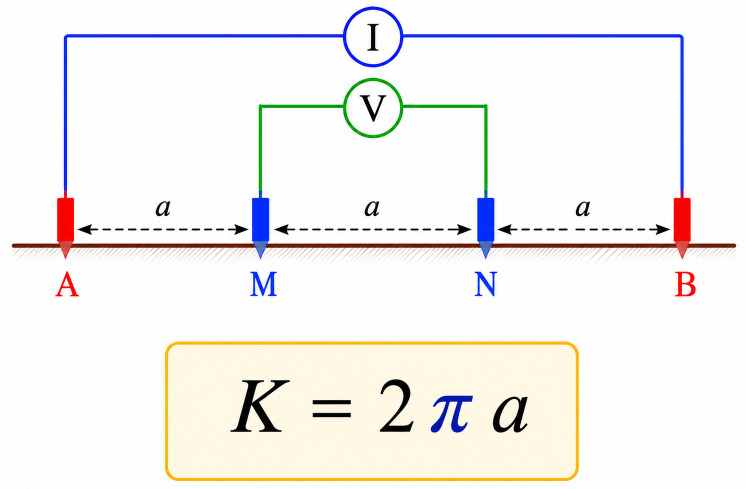
Wenner Configuration.


In this study, we conducted resistivity measurements along survey profiles of 14 m length (at the Bandung and Sumedang sites) and 24 m length (at the Subang site), adjusting profile length according to field conditions. A Wenner electrode configuration (Fig 1) was employed for all ERT surveys, as it provides a good balance of horizontal and vertical resolution for near-surface targets. The Wenner array consists of four collinear electrodes with equal spacing a between adjacent electrodes; its geometric factor is K = 2πa. Accordingly, the resistivity for a Wenner configuration is given by:

$$\begin{array}{c} \rho =2\pi a\frac{\Delta \text{V}}{\text{I}} \end{array}$$ (2)

This array was chosen because it offers strong signal strength and reliable resolution of soil layers in both horizontal and vertical directions. A variant, the Wenner–Schlumberger configuration, is known to provide moderately improved depth penetration and horizontal coverage compared to some other arrays [32]. All resistivity data in this work were collected with a portable ERT system using the Wenner array. The apparent resistivity values were calculated via Equation (1) and then inverted using RES2DINV software [32] to produce 2D resistivity sections for each profile. Inversion parameters (such as mesh discretization and regularization) were kept consistent across all profiles within a site, and the same color scale was applied to all sections from a given area to facilitate comparisons.

### 2.2. Electromagnetic induction (EMI)

**Fig 2:**
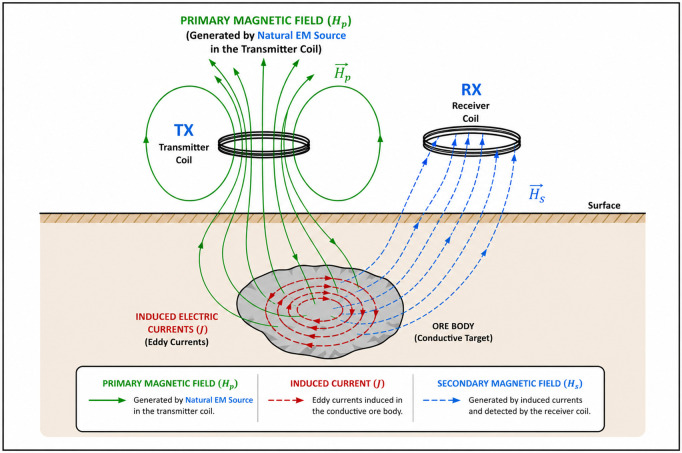
Basic principle of electromagnetic induction. The diagram illustrates that as the primary field strengthens; the secondary field assumes an opposite direction.

We also employed an electromagnetic induction (EMI) method to measure near-surface conductivity, which is the inverse of resistivity. Fig 2 shows the EMI technique (using a Geonic EM38-MK2 instrument) induces electromagnetic fields in the ground and senses the subsurface conductivity without direct contact. In an EMI survey, a transmitter coil (Tx) generates a primary oscillating magnetic field that propagates into the subsurface. This primary field induces eddy currents in conductive materials (e.g., moist or saline zones) underground, which in turn generate a secondary magnetic field. A receiver coil (Rx) measures the secondary field emerging from the ground. The magnitude of the secondary field relative to the primary field is directly related to the apparent conductivity of the subsurface when the induction number is low (i.e., for low-conductivity ground and/or low frequency) [24,29].

Under the low-induction-number approximation (valid when B is a dimensionless induction number), the secondary field response in the receiver is approximately proportional to the subsurface electrical conductivity. In this regime, one can express the bulk conductivity σ in terms of the coil spacing s, operating frequency f (with ω = 2πf), and the ratio of secondary to primary magnetic field strengths ($\frac{{\text{H}}_{\text{s}}}{{\text{H}}_{\text{p}}}$) as:


$$\begin{array}{c} \frac{{\text{H}}_{\text{s}}}{{\text{H}}_{\text{p}}}\approx \frac{\text{i}{\text{B}}^{\text{2}}}{\text{2}}=\frac{\;i\omega \mu_{\text{0}}\sigma {\text{s}}^{\text{2}}}{\text{4}}\; \end{array}$$ (3)

where σ is electrical conductivity (S/m), s is the coil spacing (m), $\mu_{\text{0}}$ is the permeability of free space, 4π x 10 − 7 Wb A^−1^ m^−1^, ω = 2πf, and f is the frequency (Hz). Modern EMI devices like the EM38-MK2 directly output apparent conductivity (σ_a_) in milliSiemens per meter (mS/m) by measuring the quadrature component of the response. The in-phase component, which is sensitive to magnetic susceptibility, is measured separately in parts per thousand (ppt). The EM38-MK2 unit we used to have two receiver coil configurations: with a 1 m transmitter-receiver spacing (vertical dipole orientation) it achieves an approximate penetration depth of ~1.5 m, and with a 0.5 m spacing it penetrates ~0.75 m (for vertical dipole). In horizontal dipole orientation, the penetration depths are roughly halved.

It is important to note that soil apparent conductivity is influenced by several factors besides water content. Saline pore fluid increases conductivity, as do higher clay content and certain mineralogy’s, whereas lower temperature can increase resistivity (decreasing conductivity). Thus, when interpreting EMI data, one must consider that a low σ_a_ (high resistivity) zone could indicate dry soil or non-saline conditions, but it could also reflect sandy texture or lower temperature, among other factors. In our study areas, however, temperature variations were minimal and the primary differences in σ_a_ are attributed to moisture and salinity differences, supported by ground truth from soil sampling.

### 2.3. Integration and merging of geophysical datasets

The primary challenge in characterizing tropical agricultural soils is bridging the gap between high-resolution vertical snapshots and field-wide lateral continuity. This study addresses this by merging the results of the primary field (ERT) with the secondary emerging field (EMI) through a standardized data transformation protocol.

#### 2.3.1. Data normalization and resistivity conversion.

To enable a direct comparison (“merging”) of the two datasets, the raw quadrature component data from the EMI (measured as apparent conductivity, $\sigma_{a}$, in mS/m) were transformed into apparent resistivity ($\rho_{a}$) values. This conversion followed the fundamental geophysical relationship:


$$\begin{array}{c} \rho_{a}=\frac{\text{1000}}{\sigma_{a}} \end{array}$$ (4)

By expressing both datasets in Ohmmeters (Ω·m), we eliminated the discrepancy between the two physical units, allowing the EMI plan-view maps to serve as a spatial “anchor” for the vertical ERT cross-sections.

#### 2.3.2. Spatial merging and scaling standardization.

The merging process involved two critical steps to ensure interpretability across the contrasting landscapes of Subang, Bandung, and Sumedang:

- Unified Color Scaling: All images were re-rendered using a consistent color scale ranging from 0 to 1,258 Ω·m. This prevents visual bias where a “blue” zone in the conductive Subang site might represent a different moisture status than a “blue” zone in the resistive Bandung site.
- Coordinate Alignment: The spatial orientation was normalized across both fields. For the merged profiles, the x-axis represents the depth (m), and the y-axis is the horizontal distance (m). This alignment allows for a “pseudo-3D” interpretation: the ERT provides the “slices” of the cake (vertical), while the EMI provides the “layers” (horizontal plan-views).

#### 2.3.3. Rationale for the integrated approach.

The rationale for merging these two fields is based on the multi-scale nature of soil water dynamics. While ERT excels at showing deep moisture pockets (up to 5 m) and stratigraphic boundaries, its linear nature leaves gaps between survey lines. The emerging EMI field fills these gaps by capturing the lateral heterogeneity of the top 1.5 m of soil. Merging these datasets allows for the identification of anisotropic moisture flow—where water might move preferentially along certain topographic gradients or textural boundaries—which would be missed by using either method in isolation. This integrated framework transforms discrete geophysical readings into a comprehensive decision-support tool for site-specific irrigation and land management.

### 2.4. Study area and data collection

The present study focuses on three agricultural areas in West Java with contrasting elevation and terrain: Subang, a low-elevation coastal plain; Bandung, a hilly upland area; and Sumedang, a moderate-elevation highland with terraced slopes. These regions not only differ in topography and climate, but also in their predominant soil textures and underlying geology. Such differences in morphology (driven by factors like climate, relief, and land use) can lead to markedly different soil water regimes and salinity profiles across the sites. The comparative analysis across Subang, Bandung, and Sumedang highlights the strong control of topography, soil texture, and geomorphology on soil moisture distribution (Table 1).

**Table 1:** Environmental and geological characteristics of the study areas in West Java, Indonesia.

Characteristic	Subang	Bandung	Sumedang
**Elevation**	Low (<50 m asl)	High (700–900 m asl)	Moderate (400–700 m asl)
**Topography**	Flat coastal plain	Steep, hilly upland	Terraced highland
**Dominant Land Use**	Irrigated rice fields	Mixed horticulture	Terraced agriculture
**Soil Texture**	Sandy loam to silty clay	Loam to sandy loam	Clay-rich subsoil
**Geological Setting**	Alluvial deposits	Volcanic products	Weathered volcanic rock
**Drainage Condition**	Poor to moderate	Rapid surface runoff	Moderate with subsurface retention

**Fig 3:**
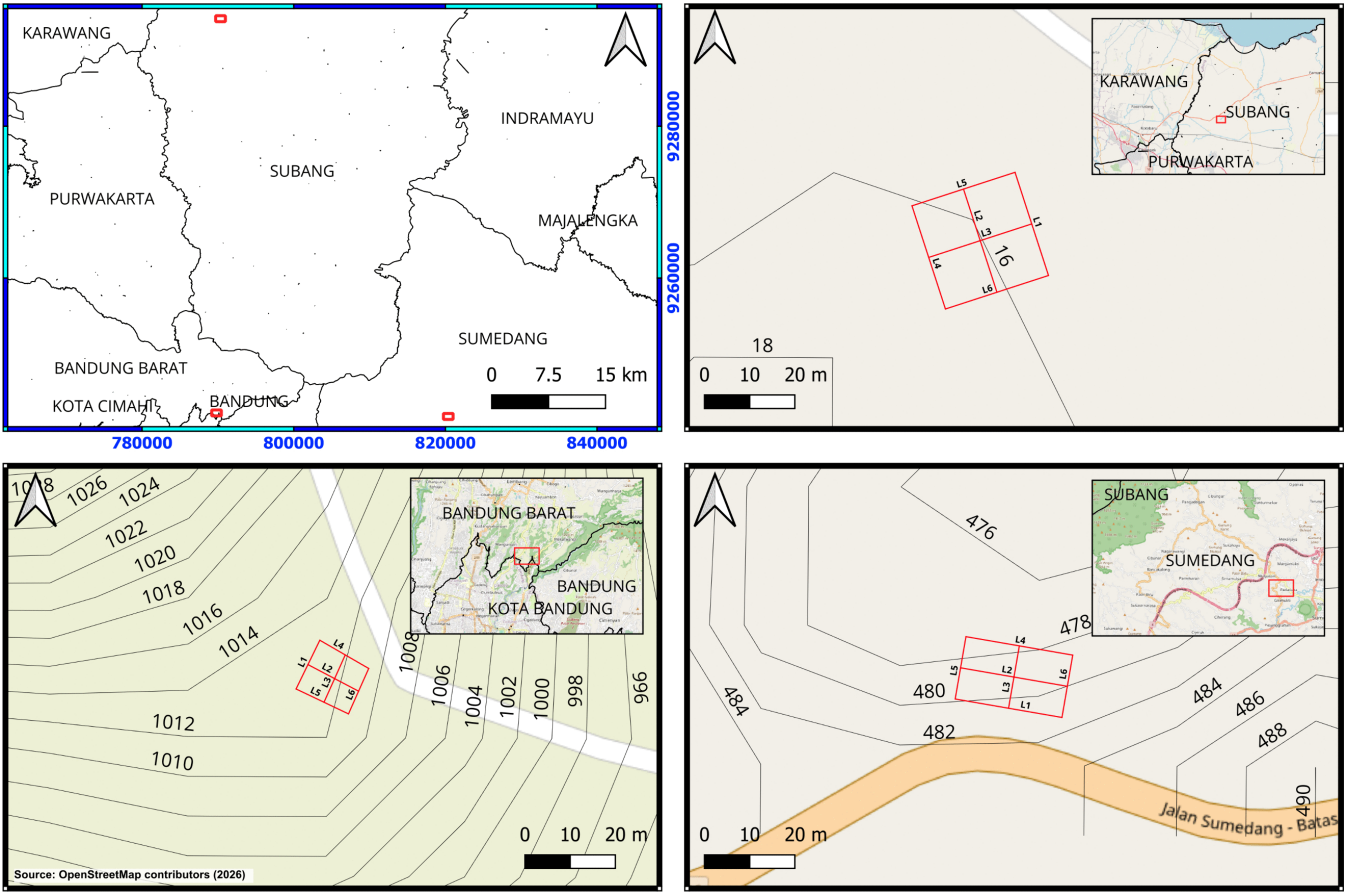
Study area and survey locations in West Java Province. (a) Provincial map showing the three study sites: Subang, Bandung, and Sumedang. Detailed maps of the (b) Subang, (c) Bandung, and (d) Sumedang areas show elevation contours (black lines) and the ERT/EMI survey profiles (red lines). North is upward in all maps. Spatial base map and road network data were obtained from OpenStreetMap (OpenStreetMap contributors, 2026) [33].

The field surveys were conducted at three locations in West Java Province, Indonesia: Subang, Bandung, and Sumedang. The measurement locations are shown in Fig 3. Subang (site 1) lies in a low coastal plain at ~16–18 m elevation, Bandung (site 2) is a hilly area at ~1011–1014 m elevation, and Sumedang (site 3) is a moderately elevated highland at ~478–480 m. These contrasting topographic settings result in different drainage characteristics: Subang’s flat terrain tends to retain water, Bandung’s steep slopes promote runoff with water accumulating in lower areas, and Sumedang’s terraced slopes have intermediate behavior. The geological background of each site also differs. The Subang area consists mainly of young alluvial deposits (clay, claystone, and associated materials), Bandung sits on ancient volcanic formations dominated by pyroclastic rocks, and Sumedang is part of a region of young volcanic products (e.g., volcanic sand and Breccia). These geologic differences contribute to the soil texture at each site: Subang’s soils are largely clay-loam, Bandung’s soils include clay in the upper layer with fine sand beneath, and Sumedang’s soils are predominantly sandy.

In Subang, the low-elevation coastal plain and fine-textured alluvial soils promote shallow water accumulation and limited drainage, resulting in relatively low resistivity and high apparent conductivity responses. Conversely, the steep upland terrain of Bandung eases rapid surface runoff and vertical infiltration, producing dry near-surface layers characterized by high resistivity values, while moisture is preferentially kept at greater depths. In contrast, the terraced highland environment of Sumedang exhibits distinct subsoil moisture retention despite dry surface conditions, as shown by low resistivity values in clay-rich layers beneath the topsoil. This behavior can be attributed to terrace morphology and reduced slope gradients, which enhance infiltration and limit lateral water loss. These contrasting responses prove that identical geophysical measurements may reflect fundamentally different hydrological processes depending on landform and soil development, underscoring the importance of site-specific interpretation in precision agriculture and irrigation planning.

At each study site, six parallel survey lines were conducted for the Electrical Resistivity Tomography (ERT) measurements. The survey lines were arranged in a rectangular grid configuration, forming a box-like layout with an added crossline positioned at the center to ensure adequate spatial coverage of a representative part of the field (Fig 3). The same profiles were subsequently surveyed using an electromagnetic induction (EMI) instrument at once after the resistivity data acquisition.

In addition to the geophysical surveys, three test pits were excavated at selected locations within each site to directly observe soil stratification, moisture conditions, and soil texture. Soil samples were collected from these pits to provide ground-truth information for confirming the geophysical interpretations. All measurements were conducted during the dry season under relatively stable weather conditions to minimize temporal variations in soil moisture during the data acquisition process.

The selection of measurement lengths for the geophysical surveys was tailored to the specific field conditions and logistical constraints of each study site. In the Subang and Sumedang areas, a profile length of 24.5 m was used to maximize spatial coverage across relatively accessible lowland and terraced terrains. In contrast, the survey length in Bandung was limited to 14.1 m due to the steeper hilly topography, dense local vegetation, and physical obstructions that constrained the available space for electrode deployment. These adjustments were necessary to ensure the highest possible data quality while working within the practical limitations of the field environment.

### 2.5. Statistical analysis

To ensure the reliability and mathematical robustness of the subsurface models, statistical validation was performed for both the ERT and EMI datasets.

#### 2.5.1. Root mean square (RMS) error.

The accuracy of the 2D Electrical Resistivity Tomography (ERT) models was evaluated using the Root Mean Square (RMS) error misfit, defined as:


$$\begin{array}{c} RMS=\sqrt{\frac{\text{1}}{N}}{\sum_{i=\text{1}}^{N}(\frac{\overset{obs}{\underset{i}{d}}--\;\overset{calc}{\underset{i}{d}}}{\overset{obs}{\underset{i}{d}}})}^{\text{2}}x\;\text{1}\text{0}\text{0}\%\; \end{array}$$ (5)

Definition of Variables

RMS: Root Mean Square error (%), standing for the overall misfit between observed and calculated data.

N: Total number of measured data points

$\overset{obs}{\underset{i}{d}}$: Observed apparent resistivity data at the i-th measurement.

$\overset{calc}{\underset{i}{d}}$: Calculated apparent resistivity data obtained from the inversion model.

i: Index of data measurement

This metric quantifies the difference between the measured apparent resistivity and the resistivity calculated from the inverted model. An inversion is considered statistically acceptable when the RMS error is below 10%, showing strong convergence. In this study:

- Subang profiles yielded the highest consistency with an average RMS error of 2.42% (range: 1.62% – 4.90%).
- Bandung profiles showed an average RMS error of 6.25% (range: 4.80% – 9.70%), reflecting the higher subsurface heterogeneity in hilly terrains.
- Sumedang profiles resulted in an average RMS error of 5.40% (range: 4.00% – 6.80%).

**Fig 4:**
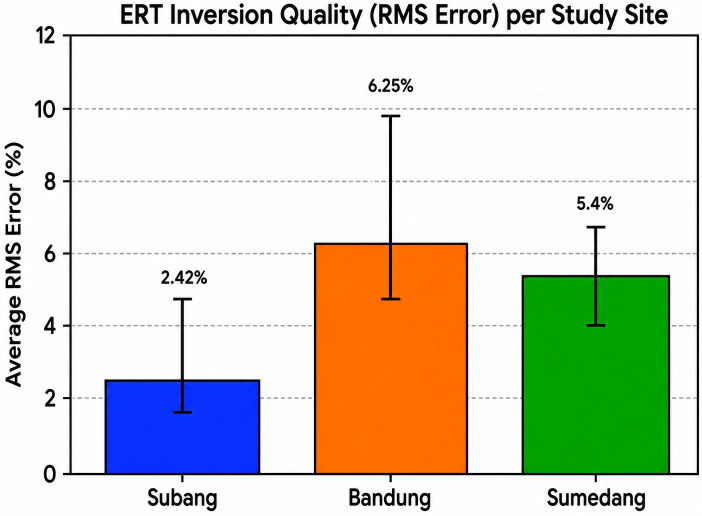
Statistical Distribution of ERT Inversion Quality (RMS Error). The bar chart illustrates the average RMS error percentages for the three study sites. The error bars are the minimum and maximum misfit recorded across the six profiles at each location.

Fig 4 describes all sites that produced an average RMS error well below the 10% threshold, confirming the high reliability of the subsurface imaging. The exceptionally low error in Subang (2.42%) is attributed to the relatively homogeneous and highly conductive clay-loam layers. The slightly higher errors in Bandung and Sumedang are representative of the complex, heterogeneous nature of volcanic-derived soils and varying topographic slopes, which introduce more noise into the geophysical signal.

#### 2.5.2. EMI data normalization and interpolation.

Electromagnetic Induction (EMI) raw data, recorded as quadrature component readings (mS/m), were converted to apparent resistivity ($\rho_{a}$) using the inverse relationship $\rho_{a}$ = $\sigma_{a}\;$to allow for direct comparison with ERT sections. Spatial variability was analyzed by interpolating the discrete measurement points using Inverse Distance Weighting (IDW). This statistical interpolation produced continuous plan-view maps at effective exploration depths of 0.35 m, 0.75 m, and 1.5 m.

#### 2.5.3. Ground-truth correlation.

The geophysical results were validated through a comparative analysis with test-pit stratigraphy. Descriptive statistics were used to verify the alignment between resistivity anomalies and the physical moisture states (desiccated vs. saturated) recorded in the pits. Qualitative correlation checks confirmed that high-resistivity zones consistently corresponded with dry topsoil layers, while low-resistivity anomalies were associated with moist subsoil horizons across all survey lines. Statistical distribution of ERT inversion quality (RMS Error) (Fig 4). The bar chart illustrates the average RMS error percentages for the three study sites. The error bars represent the minimum and maximum misfit recorded across the six profiles at each location. Analysis: All sites produced an average RMS error well below the 10% threshold, confirming the high reliability of the subsurface imaging. The exceptionally low error in Subang (2.42%) is attributed to the relatively homogeneous and highly conductive clay-loam layers. The slightly higher errors in Bandung and Sumedang are representative of the complex, heterogeneous nature of volcanic-derived soils and varying topographic slopes, which introduce more noise into the geophysical signal.

## 3. Results

In the Results section, it is recommended to explicitly present the findings for each study site—Subang, Bandung, and Sumedang—individually, for both Electrical Resistivity Tomography (ERT) and EM38 electromagnetic induction modelling. This approach will enable clearer comparison of site-specific subsurface characteristics and improve the interpretability of spatial variability across contrasting agro-landscapes.

### 3.1. Subang area (Coastal Lowland)

The Subang site is characterized by relatively flat topography and clay-rich soil. A test pit was dug to a depth of 0.55 m to examine the soil profile and moisture condition. The upper 0.25 m of the soil consisted of clay with very dry conditions, while the layer from 0.25 m down to 0.55 m was a load with noticeably higher moisture (moderately moist). Fig 4 shows the stratigraphy and appearance of the Subang test pit: a thin dry clay layer overlying a wetter loam layer.

It is important to note that the depth of the resistivity profiles and observed soil horizons varied across the three locations. These discrepancies reflect the natural geological and pedological variability inherent in West Java’s landscapes. The varying depths of the conductive zones correspond to site-specific transitions in soil-bedrock interfaces and the natural thickness of weathered layers. In Subang, the shallow water table and alluvial accumulation resulted in deeper uniform moisture profiles, while the Bandung and Sumedang sites exhibited thinner soil layers overlying more resistive volcanic parent materials. The variation in profile length between study sites was figured out by field accessibility, terrain constraints, and the spatial extent of representative agricultural plots. In Subang and Sumedang, relatively flat terrain allowed longer survey lines (~24.5 m), enabling deeper investigation depth. In contrast, the Bandung site is in a steep upland area with limited accessible space for electrode deployment, resulting in shorter survey profiles (~14.1 m). Despite these differences, electrode spacing and acquisition parameters were selected to ensure comparable vertical investigation depths for meaningful cross-site interpretation. Soil profile depth observed in test pits varied slightly across locations due to differences in excavation feasibility and soil hardness. In Bandung, the presence of compact clay limited excavation depth to approximately 0.4 m, while in Subang softer clay-loam soil allowed deeper observation. In Sumedang, natural terrace exposures provided direct observation of deeper soil profiles up to approximately 1.2 m depth.

**Fig 5:**
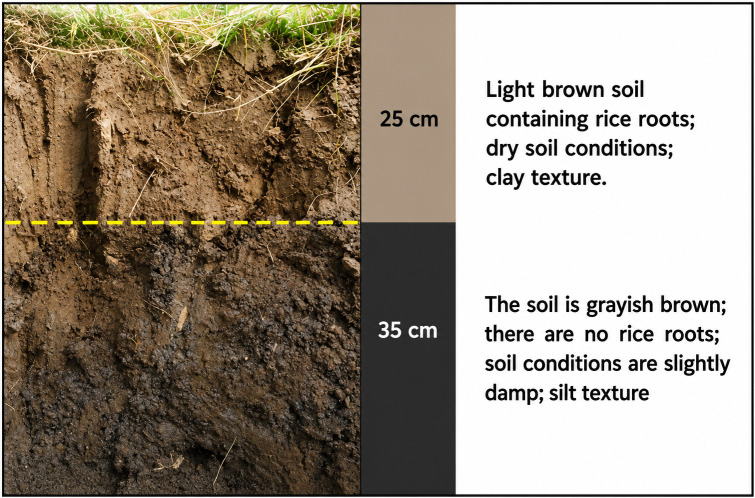
Test pit soil profile in the Subang area (pit depth 0.35 m). The top 0–25 cm is clay with very dry conditions, and 25–35 cm is loam with moist conditions. This stratification explains the resistivity contrast observed near the surface.

Six ERT profiles (L1 through L6) were measured in Subang using 0.5 m electrode spacing, each profile being ~24.5 m long. The resistivity inversion results for these profiles are compiled in Fig 5, and the inversion error statistics are given in Table 2. The ERT data was of good quality, with low Root Mean Square (RMS) misfit errors ranging from ~1.6% to 4.9%, and an average inversion error of 2.42% for the Subang dataset (Table 2). The resistivity cross-sections in Fig 5 use a consistent color scale for all lines in Subang, facilitating comparison. Broadly, the Subang ERT sections reveal a dominance of low resistivity values near the surface. The resistivity values are illustrated by a blue green–red color scale, where blue indicates low resistivity (on the order of ~1.7 to 10 Ω·m), green indicates an intermediate resistivity (around 10–43 Ω·m), and yellow to red indicates higher resistivity (tens to a few hundred Ω·m). These resistivity variations correspond to different soil conditions and water content along the profiles.

**Table 2:** ERT survey line details for Subang area (electrode spacing 0.5 m).

Line	Length (m)	RMS Error (%)
**L1**	24.5	2.00
**L2**	24.5	1.85
**L3**	24.5	2.00
**L4**	24.5	2.20
**L5**	24.5	1.62
**L6**	24.5	4.90

In the Subang ERT sections (Fig 5), the uppermost layer shows relatively higher resistivity in some places (red hues), corresponding to the dry clayey topsoil observed in the test pit. Beneath this, extensive blue areas (low resistivity) appear, indicating zones of higher moisture content. Indeed, field observations confirm that only a thin surface layer (~0.2 m thick) was dry, underlain by wetter soil. The dry surface yields higher resistivity near the top, while the increase in moisture below ~0.3 m depth causes a sharp drop in resistivity (blue anomalies). Line L6 is a slight exception, showing a small low-resistivity anomaly even at the surface, perhaps due to localized conditions. Overall, the ERT suggests that beyond about 0.25–0.3 m depth, the Subang soil is uniformly moist, which agrees with our test pit that encountered moist loam below 0.25 m. We hypothesize that the high-water content extends downward from this depth, given the consistently low resistivity observed throughout the deeper part of the sections.

**Fig 6:**
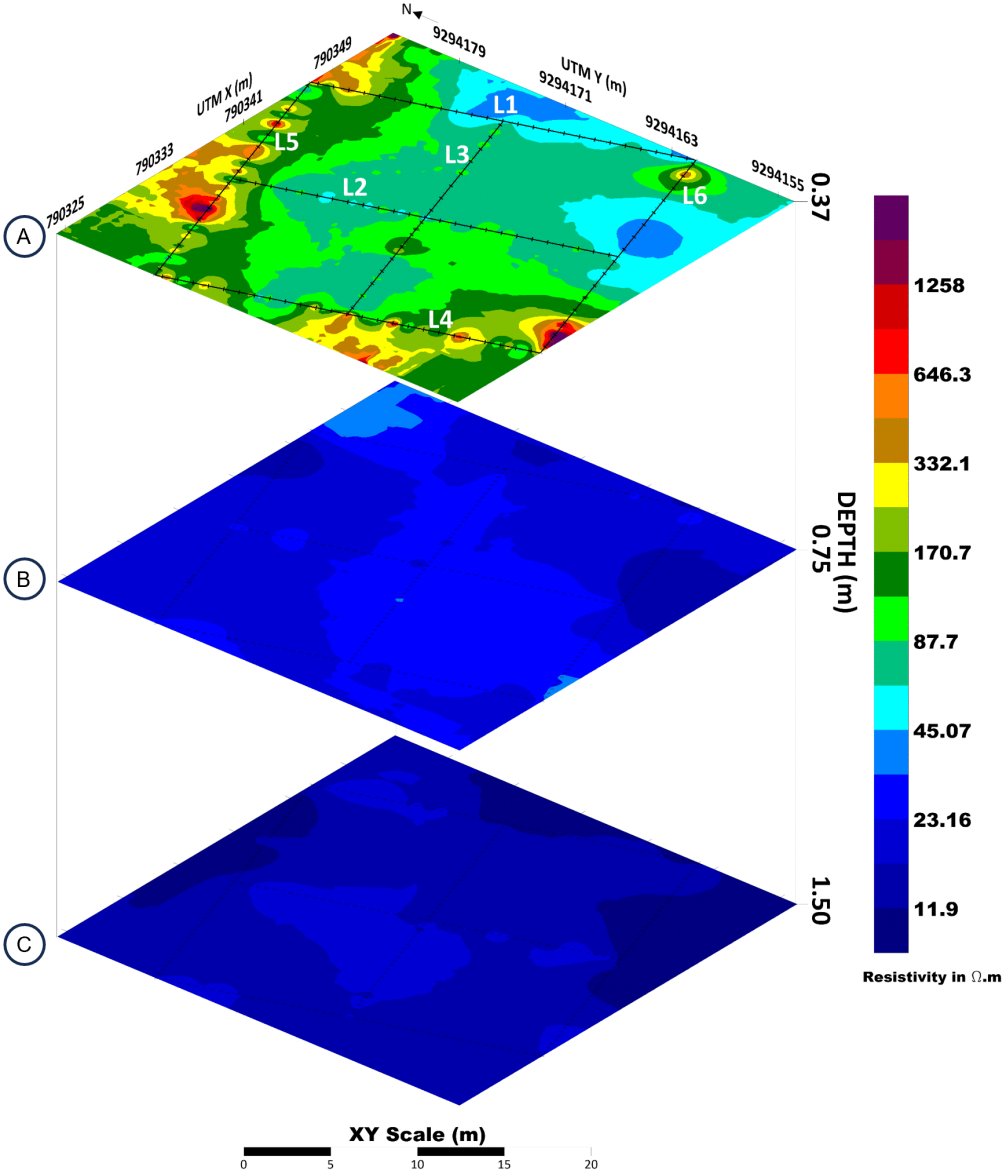
Plan-view resistivity distribution maps from EMI (EM38) measurements in the Subang area at different effective exploration depths: (A) ~0.35 m, (B) ~0.75 m, and (C) ~1.5 m. High resistivity (red) at 0.35 m highlights dry compacted surface soil. Low resistivity (blue) dominates at 0.75–1.5 m depths, indicating higher moisture content in the subsoil, which corresponds with the ERT results and field observations.

**Fig 7:**
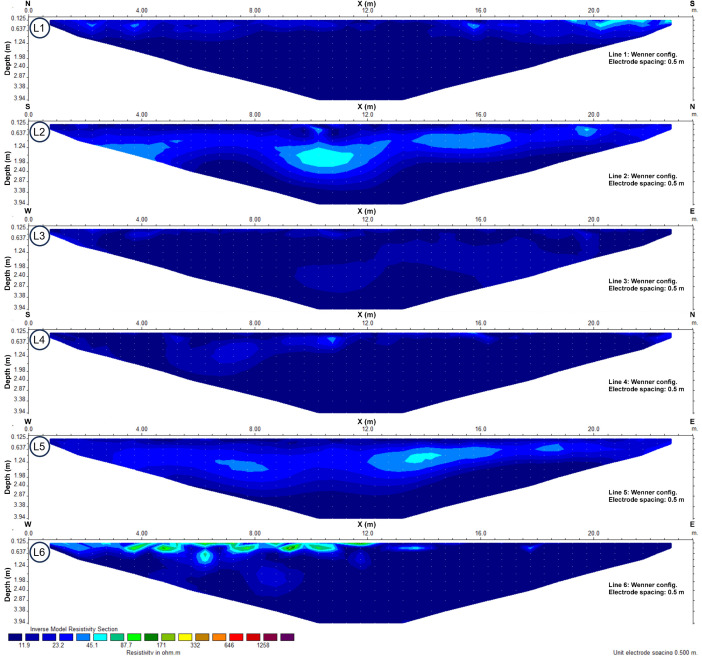
ERT resistivity sections for the Subang site (profiles L1 through L6, top to bottom). All sections use the same color scale (blue = low resistivity, red = high resistivity) with values calibrated as shown. Near-surface dry clay yields higher resistivities (warm colors), while deeper moist zones appear as low-resistivity anomalies (cool colors). The thin dry layer and extensive moist subsoil are consistent across most profiles, except L6 shows a localized anomaly near surface.

In addition to resistivity imaging, we performed EMI measurements along the same lines in Subang to map apparent electrical conductivity of the soil. The raw EMI data (quadrature component) were recorded in mS/m and later converted to apparent resistivity (Ω·m) for ease of comparison with ERT results (using ρ = 1/σ, with appropriate unit conversions). We interpolated the EMI readings to create plan-view resistivity maps (“slicing” the data) at representative investigation depths of approximately 0.35 m, 0.75 m, and 1.5 m. Fig 6 shows these resistivity distribution maps for the Subang site derived from the EM38 data. To maintain consistency, the color scale for these maps was adjusted to match the color scale of the ERT sections (Fig 7).

The plan-view resistivity distribution maps derived from EMI measurements (Fig 6) highlight the spatial variability of shallow soil moisture across the Subang agricultural field. These maps provide critical spatial continuity, complementing the vertical snapshots provided by ERT. To ensure consistency, all maps use a standardized resistivity Ω·m) scale of 0–1258 Ω·m, with the x-axis representing depth (m) and the y-axis representing distance (m).Shallow Subsurface Conditions (0.35 m Depth)At the shallowest effective exploration depth (~0.35 m; Fig 6A), the Subang site is characterized by widespread high resistivity (red-orange colors). These high values correspond to the dry, compacted topsoil observed in the test pit (0–0.25 m). As noted by Widodo [29], the combination of surface dedication in the tropical coastal climate and soil compaction from agricultural machinery leads to low conductivity (high resistivity) in this upper crust. This resistive “cap” acts as a barrier to immediate infiltration, as evidenced by the lack of moisture detected in the shallowest EMI slice.

Subsoil Moisture and Conductivity (0.75 m and 1.5 m Depths)

In contrast, the deeper slices at 0.75 m and 1.5 m (Fig 6B,C) reveal a predominant shift toward low resistivity (blue-green colors). This trend is consistent with significantly higher moisture content in depth. Specifically:

- At 0.75 m: The transition to lower resistivity is the moist loam layer encountered in the test pit below the clay cap.
- At 1.5 m: The maps are dominated by deep blue anomalies Ω·m), showing a uniformly saturated and highly conductive zone.

These EMI results align well with the ERT findings: the surface of Subang’s soil is relatively dry and resistive due to the thin clay cap and compaction, while the subsoil holds substantial moisture and is significantly more conductive. Furthermore, the pervasive nature of the low-resistivity zone at 1.5 m suggests that moisture accumulation is not localized but is a field-wide characteristic of this coastal lowland, likely influenced by a shallow water table and elevated soil salinity. This integrated approach confirms that while the surface appears parched, the underlying agro-landscape keeps high water reserves, a critical factor for site-specific irrigation management.

### 3.2. Bandung area (Hilly upland)

**Fig 8:**
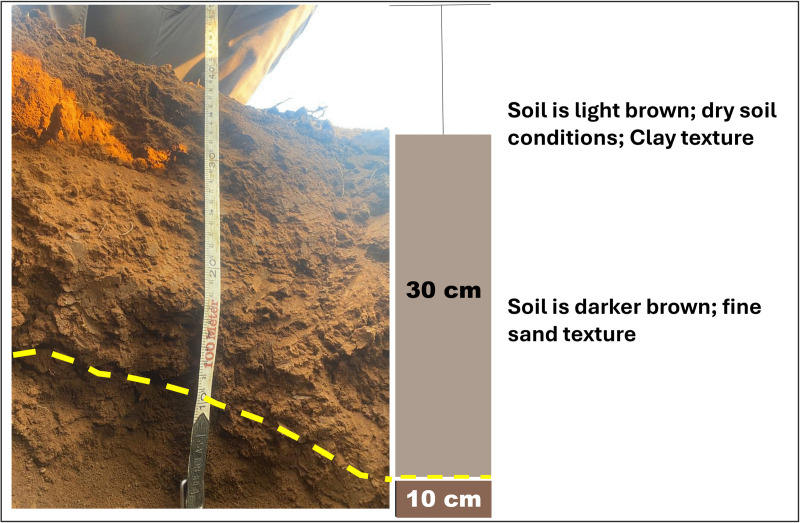
Test pit soil profile in the Bandung area (pit depth 0.4 m). The upper ~30 cm is clay with extremely dry conditions (hard, desiccated soil), while 30–40 cm depth is very fine sand that is moist. This indicates that rainfall infiltrated and was retained at depth in the sand, while the clay surface dried out.

The Bandung site has a higher elevation and steeper terrain than Subang, with soils influenced by volcanic parent material. A test pit in Bandung reached 0.4 m depth and exposed two distinct layers: from 0 to ~0.3 m the soil is clayey and extremely dry, while from ~0.3 to 0.4 m it transitions to a very fine sand that was observed to be moist. This suggests that near-surface water had largely evaporated in the dry season, and moisture was present only in the slightly deeper sandy layer. Fig 8 shows the Bandung test pit and confirms the parched condition of the topsoil at the time of measurement.

We conducted six ERT profiles in Bandung (L1–L6) with 0.3 m electrode spacing, each profile being ~14.1 m long (covering a smaller area due to field constraints in the hilly terrain). The inversion results for these ERT lines are presented in Fig 8, with their RMS errors given in Table 3. The inversions had acceptable misfits (error range ~4.8%–9.7%, average ~6.25%). The resistivity cross-sections for Bandung (Fig 8) reveal a markedly different resistivity distribution compared to Subang. Here, the near-surface resistivity values are generally higher overall, which is consistent with the observed dryness of the topsoil. In the color scale applied (blue to red), blue still represents the lowest resistivities (~7–51 Ω·m in Bandung’s case) and red the highest (up to ~1270 Ω·m). The Bandung ERT sections show extensive high-resistivity zones (green to red colors) near the surface across most profiles, reflecting the very low moisture content in the top ~ 0.3 m of clayey soil.

**Table 3:** ERT survey line details for Bandung area (electrode spacing 0.3 m).

Line	Length (m)	RMS Error (%)
**L1**	14.1	6.8
**L2**	14.1	9.7
**L3**	14.1	5.1
**L4**	14.1	4.8
**L5**	14.1	5.4
**L6**	14.1	5.7

**Fig 9:**
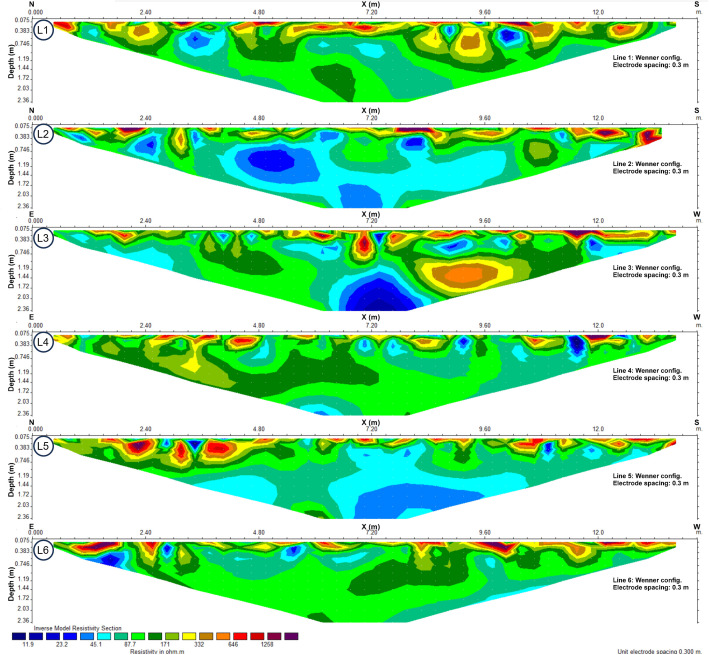
ERT resistivity sections for the Bandung site (L1–L6). All sections use a consistent color scale (blue ≈ 8–51 Ω·m, red ≈ 51–1271 Ω·m for Bandung). The hilly Bandung site shows predominantly high resistivity (green-red colors) near the surface, corresponding to the very dry clay topsoil. Some profiles (e.g., L2, L3, L5) show low-resistivity (blue) pockets below ~0.5 m, suggesting zones of higher moisture content at depth (like in lower-lying portions of the field). Overall, Bandung’s resistivity values span a wide range, reflecting the heterogeneous soil moisture and texture conditions.

At greater depths (below ~0.5 m), the Bandung profiles begin to show pockets of lower resistivity (blue zones) in some lines, particularly L2, L3, and L5 (Fig 9). These low-resistivity anomalies at depth suggest zones of higher water content—likely the fine sand layer retaining moisture as indicated by the test pit. Notably, lines L2, L3, and L5 are in slightly lower-lying parts of the site (small depressions or foot-slope positions), which could accumulate more moisture relative to the other lines on higher ground. This observation leads to a hypothesis that below about 0.5 m depth, the Bandung site contains higher water content in pockets, potentially extending down to ~2–3 m depth in the subsurface. The overall resistivity range in Bandung (roughly 8 Ω·m to over 1000 Ω·m) is much broader than in Subang, indicating more heterogeneity – likely due to the combination of extremely dry soil in some areas and the presence of more resistive sandy or rocky material, along with localized moisture at depth.

**Fig 10:**
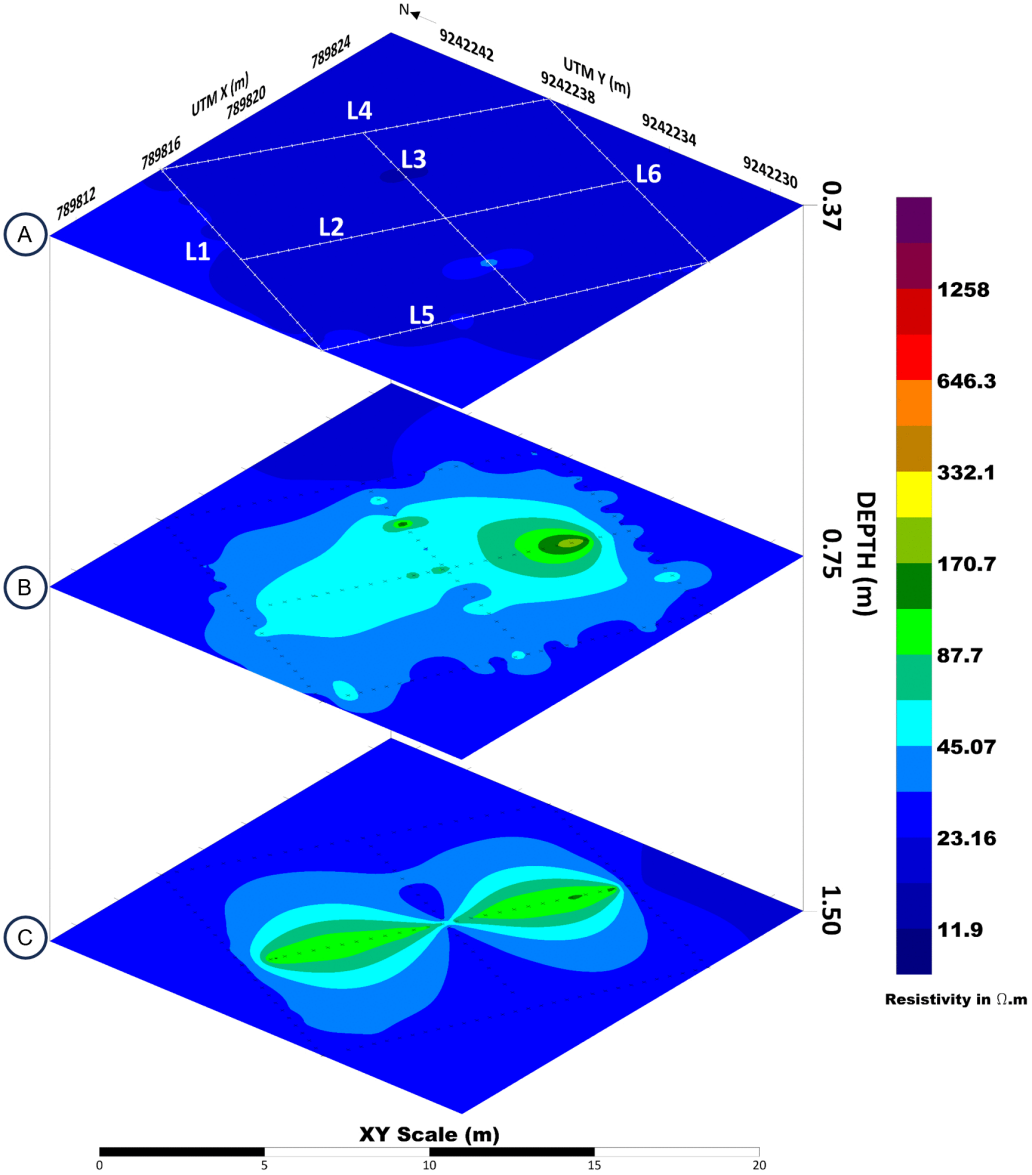
Plan-view resistivity distribution maps from EMI measurements in the Bandung area at depths of approximately (A) 0.33 m, (B) 0.75 m, and (C) 1.5 m. In the shallow slice (A), parts of the surface show low resistivity (blue) even though the soil was dry – this is attributed to the clayey soil’s inherent conductivity and any residual salinity. At deeper slices (B, C), higher resistivity zones emerge (green, yellow), corresponding to the sand layer with lower moisture. These EMI maps corroborate the ERT findings, capturing the dry surface clay and the varied materials at depth.

**Following the ERT surveys, EMI measurements were conducted to map the spatial distribution of apparent resistivity across the Bandung site at effective exploration depths of approximately 0.33 m, 0.75 m, and 1.5 m (** **Fig 10** **). To support analytical consistency, all plan-view maps use a standardized resistivity scale of 0–1258 Ω·m, with the x-axis being depth (m) and the y-axis being distance (m). Shallow Surface Conditions (~0.33 m Depth)**

In contrast to the Subang site, the shallow EMI map for Bandung (Fig 10) reveals predominantly low resistivity (high conductivity) at the surface. This result is initially surprising given that the topsoil was visibly dry and desiccated during the survey. However, this phenomenon is explained by the specific soil composition of the Bandung site:

- Mineralogical Influence: The surface consists of clay, which supports moderate conductivity even when dry due to residual bound water and a high Cation Exchange Capacity (CEC).
- Agricultural Factors: Current agricultural practices, including the application of fertilizers or the presence of organic matter, likely contribute to the observed conductivity.
- Conductivity Values: While the soil appeared dry, its apparent conductivity remained in the range of tens of mS/m, preventing the high resistivity readings typically expected from completely dry sand.

**Subsurface Transitions (0.75 m and 1.5 m Depths)**

At deeper slices (0.75 m and 1.5 m; Fig 10B, C), the Bandung site shows higher resistivity zones compared to the surface. While seemingly counterintuitive, this pattern reflects a significant vertical transition in soil texture:

- Textural Shift: The increasing resistivity at depth corresponds to the transition from surface clay to a fine sand layer (observed below ~0.3 m in the test pit). Sand inherently has higher resistivity than clay, especially when it is only partially saturated or compacted.
- Methodological Consistency: These EMI findings are highly consistent with the ERT sections, which also showed higher resistivity values starting at ~0.3 m where the sandy material was present.

Topographic Controls and Moisture Retention

In summary, both ERT and EMI data consistently show that the immediate surface in Bandung has lower resistivity due to its clayey nature and residual minerals, while resistivity increases as the sensors probe the underlying sandy horizon. The overall results highlight a very dry soil profile, with the steepest desiccation at the surface clay layer and limited moisture retained within the sandy sub-layer at 0.3–0.5 m depth. Furthermore, the hilly terrain drives an uneven distribution of moisture; lower-lying portions of the field function as accumulation zones, manifesting as localized low-resistivity anomalies in both geophysical datasets.

### 3.3. Sumedang area (Highland Terrace)

**Fig 11:**
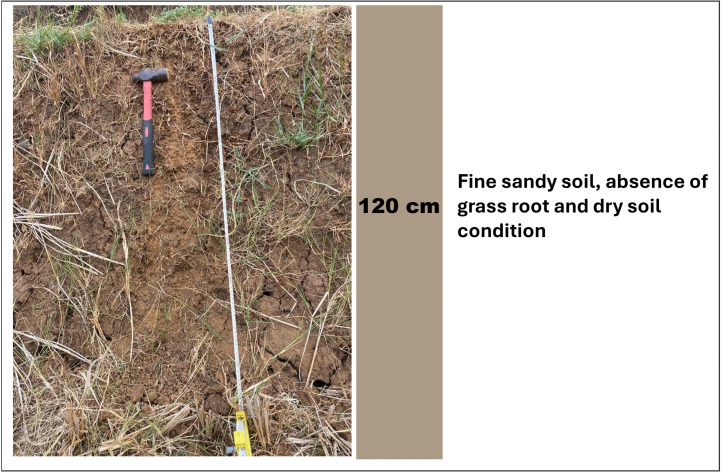
Exposed soil outcrops on a terraced farmland in the Sumedang area. The profile (to ~1.2 m depth) consists of homogeneous fine sand, which was observed in a dry state at the exposed face. The dryness of this outer soil likely results from evaporation on the terrace face, whereas soil further inward may retain more moisture.

The Sumedang site is an upland area characterized by terraced farmland on slopes and predominantly sandy soil. Unlike Subang and Bandung, an exposed soil section (outcrop) was available in Sumedang at a terrace cut, allowing direct observation of the soil profile to about 1.2 m depth. This exposure (Fig 11) revealed that the soil is homogeneous fine sand throughout, and at the time of study the exposed soil appeared dry. The absence of clay in the soil profile means that water retention characteristics in Sumedang differ from the other sites – sandy soils drain quickly and can become dry unless there is frequent rainfall or irrigation.

### 3.4. Soil textural and mineralogical composition

The physical and chemical properties of the soil at each study site—Subang, Bandung, and Sumedang—were characterized to provide a baseline for interpreting the geophysical survey results. These properties, including soil texture and mineralogy, are summarized in Table 4.

**Table 4:** Soil Textural and Mineralogical Composition of the Study Sites.

Study Site	Predominant Soil Texture	Primary Mineral Composition	Dominant Clay Minerals
**Subang (Coastal Lowland)**	Clay Loam	Quartz, Feldspar, Halite (trace)	Montmorillonite, Illite
**Bandung (Hilly Upland)**	Sandy Loam	Quartz, Cristobalite, Feldspar	Kaolinite
**Sumedang (Terraced Highland)**	Silty Clay	Halloysite, Hematite, Goethite	Halloysite, Kaolinite
**Study Site**	Predominant Soil Texture	Primary Mineral Composition	Dominant Clay Minerals
**Subang (Coastal Lowland)**	Clay Loam	Quartz, Feldspar, Halite (trace)	Montmorillonite, Illite
**Bandung (Hilly Upland)**	Sandy Loam	Quartz, Cristobalite, Feldspar	Kaolinite

The soil in Subang is classified as Clay Loam, originating primarily from alluvial and marine deposits. The mineralogy is dominated by expanding lattice clays, specifically Montmorillonite (Smectite group) and Illite. These minerals are known for their high specific surface area and high Cation Exchange Capacity (CEC). The presence of these active clay minerals, combined with residual salinity (trace Halite) from the coastal environment, eases high electrical conductivity. This geochemical makeup directly explains the exceptionally low resistivity values (1.7–30 Ω·m) saw across all ERT profiles in this region.

In the hilly terrain of Bandung, the soil is primarily a Sandy Loam developed from the weathered volcanic products of the Tangkuban Perahu complex. The mineral composition is dominated by resistive silicate minerals, namely Quartz and Feldspar, with Kaolinite serving as the primary clay constituent. Unlike the clays found in Subang, Kaolinite is a non-expanding clay with a relatively low CEC. The high sand content promotes rapid drainage and high macro-porosity. These factors, coupled with the inherently resistive nature of the silicate framework, result in the significantly higher resistivity range (70–300 Ω·m) characteristic of the Bandung site.

The Sumedang site features a Silty Clay texture derived from older volcanic ash deposits. The mineralogy is characterized by Halloysite and a high concentration of iron oxides, such as Hematite and Goethite, which impart a typical reddish-brown volcanic hue to the soil. This specific mineralogical composition allows for moderate CEC and high microporosity, enabling the subsoil to retain moisture effectively even during dry periods. The presence of these clay minerals and iron oxides supports the intermediate resistivity observed, where moisture is retained in deeper layers even when the surface appears desiccated due to terrace-face evaporation.

**Fig 12:**
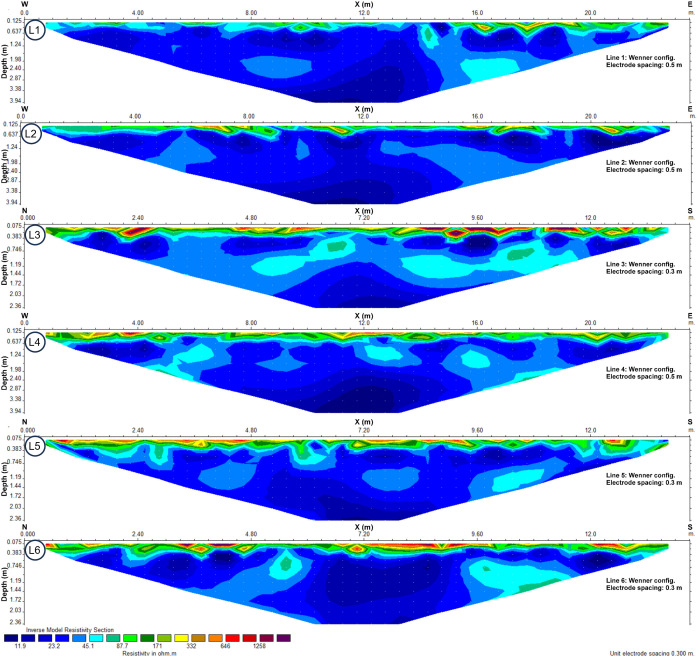
ERT resistivity sections for the Sumedang site (L1–L6). Blue colors represent low resistivity (~8–30 Ω·m) and red colors high resistivity (~114–817 Ω·m) in Sumedang. The near surface is mostly moderate to high resistivity (green to red), reflecting dry sandy soil at the exposed terrace surfaces. However, at depths >0.5 m, patches of low resistivity (blue) appear, suggesting zones of higher moisture content in the subsurface. The outermost parts of the profiles (corresponding to terrace edges) remain resistive due to evaporation and drying, while interior portions at depth are more conductive (moist).

Six ERT survey lines were acquired at the Sumedang site (Table 5). Three longer profiles (L1–L3), each approximately 24.5 m in length, were measured using an electrode spacing of 0.5 m, while three shorter profiles (L4–L6), each approximately 14.1 m long, were measured with a spacing of 0.3 m. This configuration was selected to accommodate local terrain constraints while also improving depth resolution for certain profiles. The inverted resistivity models for the Sumedang site are presented in Fig 12, with the corresponding inversion error statistics summarized in Table 3. The average root mean square (RMS) inversion error is approximately 5.4%, which is comparable to the values obtained at the other study sites and shows a reliable inversion result.

**Table 5:** ERT survey line details for Sumedang area (L1–L3 with 0.5 m spacing, L4–L6 with 0.3 m spacing).

Line	Length (m)	RMS Error (%)
**L1**	24.5	4.1
**L2**	24.5	4.0
**L3**	24.5	4.60
**L4**	24.5	6.80
**L5**	24.5	6.80
**L6**	24.5	6.10

The resistivity cross-sections for Sumedang (Fig 12) are dominated by moderate to high resistivity values, with limited occurrences of low-resistivity zones near the surface. This pattern contrasts with the Subang site and reflects the relatively dry conditions of the sandy surface soils in Sumedang. In the adopted color scale, blue is the lowest resistivity values (approximately 8–30 Ω·m), while red shows the highest resistivity values (approximately 114–817 Ω·m). Most of the near-surface layers in the Sumedang profiles are represented by green to yellow colors, corresponding to moderate resistivity values ranging from approximately 60–100 Ω·m or higher. These values are consistent with relatively dry sandy soils. However, localized blue zones appear at depths greater than approximately 0.5 m, which are likely to represent areas with higher moisture content. These low-resistivity anomalies may show zones where infiltrating water accumulates in deeper soil layers, potentially associated with reduced permeability horizons or proximity to the local groundwater table.

Field observations support this interpretation: the exposed terrace surface (outermost part of the field) was very dry at the time, which correlates with high resistivity near the surface in the ERT. Meanwhile, we suspect that further into the slope or beneath the terrace, moisture is retained, which could explain the low-resistivity zones at depth. The outermost terrace soils experience more direct sun and wind exposure, leading to evaporation and drier conditions, whereas soil a bit further from the exposed face might hold more moisture. Thus, one hypothesis is that the evaporation at the step-like terrace face causes the outer soil to be drier (and more resistive), while the inner soil (away from the face) remains relatively moister. This is evidenced by Fig 12 sections where near-surface resistivity is high at the edges corresponding to terrace faces, but lower resistivity appears a short distance inward at depth.

**Fig 13:**
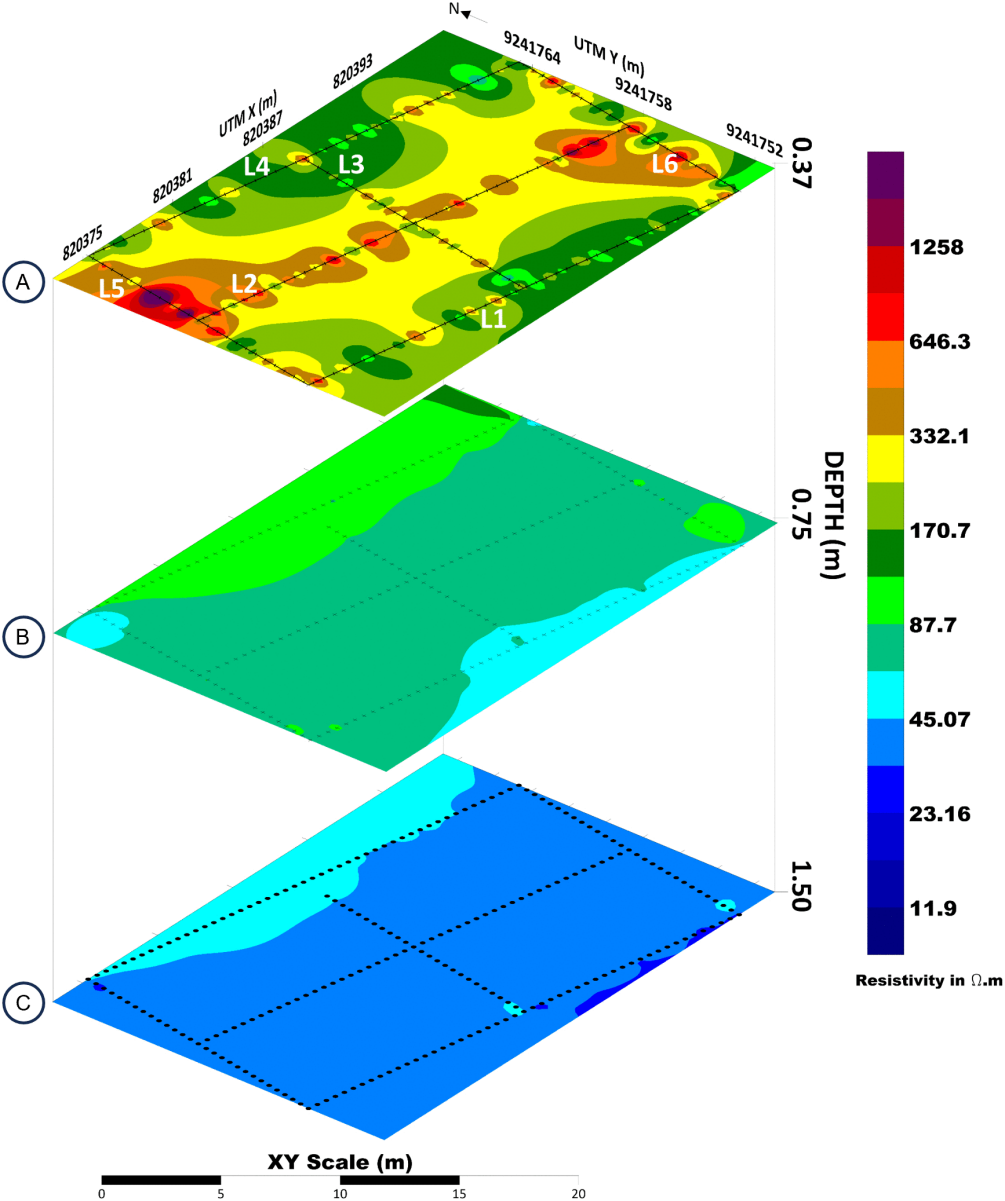
Plan-view resistivity distribution maps from EMI measurements in the Sumedang area at approximate depths of (A) 0.35 m, (B) 0.75 m, and (C) 1.5 m. Near the surface (A), high resistivity (red) dominates, indicating dry, compacted sand at the exposed terrace surfaces (low conductivity). At deeper levels (B, C), resistivity is lower (blue green), revealing the presence of moisture in the soil away from the exposed face and at depth, consistent with the conductive nature of water in the subsurface.

EMI surveys in Sumedang were conducted similarly, and Fig 13 shows the EMI-derived resistivity maps at ~0.35 m, 0.75 m, and 1.5 m depths. At the shallow slice (0.35 m, Fig 13A), high resistivity (red) is prevalent at the surface, indicating very dry conditions in the sandy topsoil (as expected from the field observation of the dry exposure). This dry, compacted sand yields low conductivity (high resistivity) values at the surface, analogous to the effect seen in Subang’s compacted dry layer (though here it’s sand instead of clay). At deeper slices (0.75 m and 1.5 m, Fig 13B, C), the maps show lower resistivity (< 90 Ω·m, blue-green colors) in many areas of Sumedang, which aligns with the idea that moisture content increases with depth or is retained in less exposed parts of the soil. Water is conductive, so the presence of moisture at depth in the sand would reduce resistivity. The EMI maps thus reinforce the ERT interpretation: the surface of Sumedang’s soil is dry and resistive, but at greater depth (or further into the terraces) the soil is relatively wetter and more conductive, despite the overall sandy texture.

In summary, the Sumedang site shows that even in a coarse-textured soil which drains quickly, there can be significant moisture at depth that is not apparent from surface conditions. The geophysical data captured these differences, with ERT and EMI both indicating drier conditions at the exposed surface and wetter zones below about 0.5–1 m.

## 4. Discussion

- **Resistivity Survey Results – Moisture Distribution Patterns**

**Fig 14:**
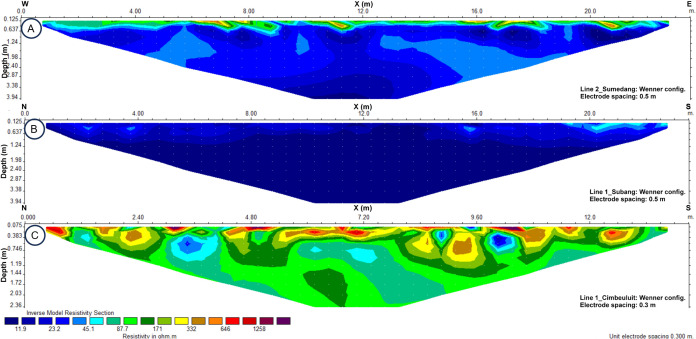
Resistivity cross-section L1 in (top to bottom) Subang area (A), Sumedang area (B), and Bandung area (C) using Wenner configuration.

The ERT measurements reveal clear differences in subsurface resistivity (and thus inferred moisture content) among the three study areas (Fig 14). The Subang (coastal lowland) site is dominated by very low resistivity values – approximately 1.7 to 30 Ω·m in the upper 2–3 m – indicating that the soil is highly conductive. Such low resistivity corresponds to high moisture content and/or elevated salinity, as pore water with dissolved ions greatly enhances conductivity. Indeed, the presence of near-surface groundwater and possibly brackish soil water in Subang’s flat coastal plain leads to uniformly moist, conductive conditions. By contrast, the Bandung (hilly) site shows predominantly moderate to high resistivity values in its soil profile, ranging roughly from 70 up to 300 Ω·m. These higher resistivities reflect much drier soil conditions and lower salinity overall in the upland Bandung terrain. The Sumedang (highland terraced) site exhibits an intermediate behavior: resistivities in some shallow zones are low (on the order of 8–30 Ω·m, akin to Subang), but large portions of the profile show moderate values up to ~200 Ω·m. The lowest resistivities in Sumedang were typically measured at deeper layers (below about 0.5 m), suggesting pockets of higher moisture content at depth, whereas the near surface in Sumedang was somewhat drier (higher resistivity) than in Subang.

These resistivity patterns can be interpreted in terms of each location’s hydrology and soil characteristics. Subang’s consistently low resistivity indicates that its clay-loam soil retains significant moisture throughout the profile; field observations confirmed that even the surface layer in Subang was relatively moist and rich in fine-textured clay. Sumedang also shows low resistivity (high moisture) zones, but notably these occur below the surface. The test pit in Sumedang revealed a drier sandy loam topsoil overlying wetter clay-rich soil at depth, which explains why the lowest resistivity values (indicating wet soil) were recorded a bit deeper in the profile. Bandung, on the other hand, had the highest resistivity readings, consistent with very low moisture content in its soil during the survey. The Bandung pit showed that the top ~ 30 cm of soil (a clayey layer) was extremely dry, and only a thin layer of slightly moist sand was encountered at ~30–40 cm depth before reaching hard substrate. Therefore, the resistivity data clearly reflects these conditions: Bandung’s hillslope soils were mostly dry (high resistivity), whereas Subang’s flat coastal soils were pervasively moist (very low resistivity), with Sumedang’s terraced land falling in between. Furthermore, the fact that Subang’s near-surface resistivities are as low as those at depth implies some degree of salinity in the coastal soil (since dissolved salts would maintain high conductivity even in surface layers that experience evaporation). This aligns with the expectation that coastal lowland soil, influenced by marine or estuarine processes, can have elevated salt content and water tables, keeping resistivity low. Consistently, Subang’s pit exhibited signs of salt presence (white efflorescence) and wet clay even at 25 cm depth, whereas Bandung’s pit was completely dry at that depth.

- **Electromagnetic Induction (EMI) Results – Vertical Moisture Profiles**

**Fig 15:**
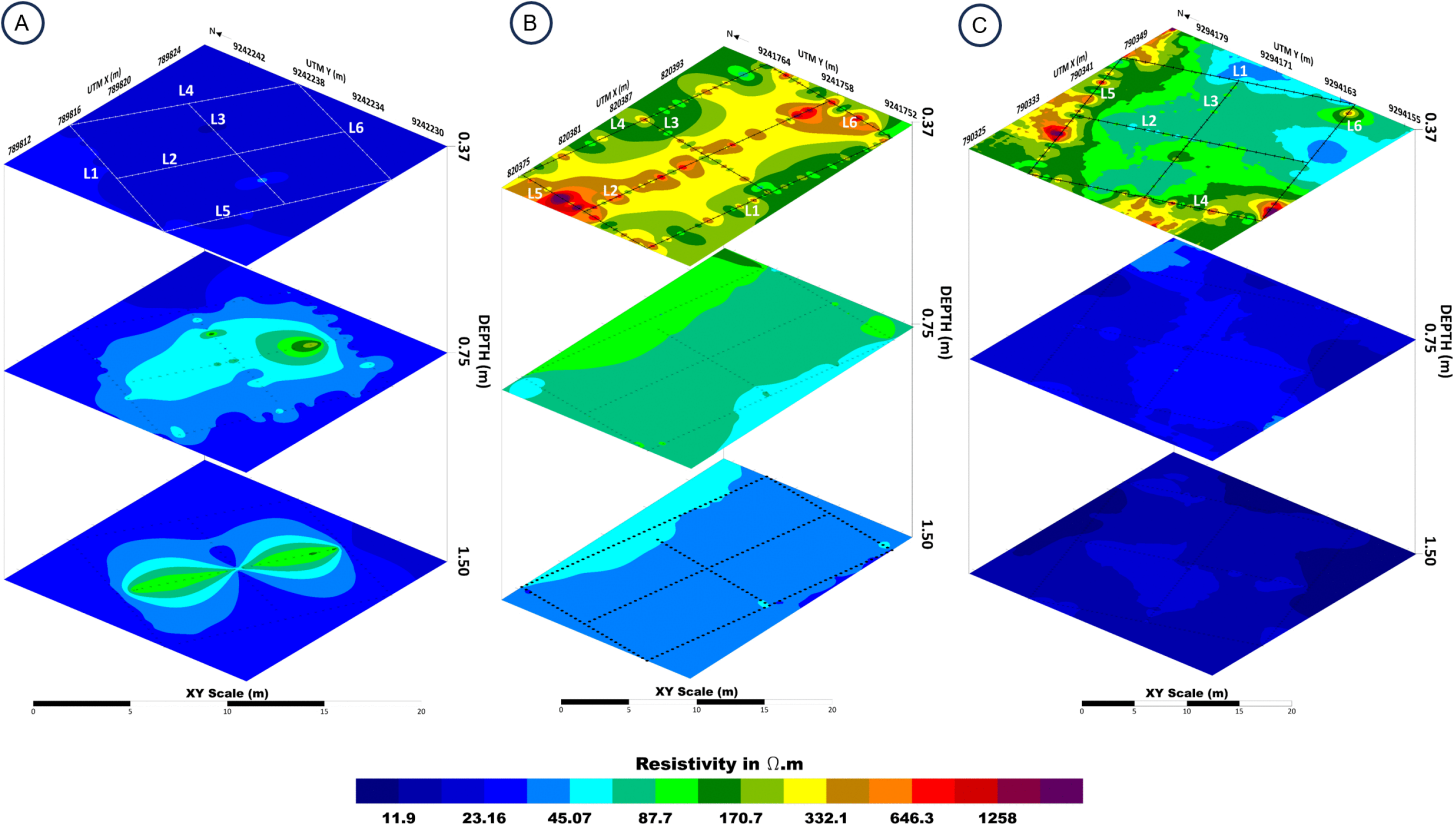
Map of electromagnetic induction measurement resistivity distribution on (left to right) Subang area (A), Bandung (B), and Sumedang (C) at varying depths.

The EMI surveys (Fig 15), which measure apparent conductivity at different effective penetration depths, complement the ERT findings by providing laterally extensive maps of moisture distribution at specific depth slices. For interpretation, the EMI conductivity data were converted to equivalent resistivity values for direct comparison with ERT results. At a shallow investigation depth (approximately 0.3–0.4 m), the Subang and Sumedang areas both show high resistivity readings (i.e., low apparent conductivity) in the EMI data, whereas Bandung shows much lower resistivity (higher conductivity) at the same depth. This indicates that, at the time of measurement, the topsoil (~0.3 m) in Bandung retained more moisture than the topsoil in Subang and Sumedang. One plausible reason is that Bandung’s clay-rich topsoil, although dry at the surface when sampled, may have absorbed some rainfall or dew and temporarily held moisture near the surface (the site is at higher elevation with cooler, misty mornings).

In contrast, Subang’s and Sumedang’s exposed soils likely experienced greater evaporation, leaving the shallow layer relatively dry despite abundant moisture below. Indeed, EMI maps for the 0.75 m and 1.5 m depth slices show the opposite pattern: at these deeper levels, Subang and Sumedang exhibit very low resistivity (indicative of high moisture content), whereas Bandung’s deeper soil shows higher resistivity. In other words, moisture in Bandung was concentrated near the surface (and did not penetrate deeply, possibly due to a coarse sand layer limiting downward retention), while in Subang and Sumedang, moisture was concentrated at deeper layers (with drier soil above). These observations are consistent with the different infiltration and drainage characteristics of the soils: Bandung’s thin topsoil overlying sand allows rapid percolation with little water retention at depth (any deep moisture may drain further down the slope or be very localized), whereas Subang’s flatter terrain and heavier soil texture facilitate the accumulation of water in the subsoil. Sumedang’s terraced slopes act somewhat like stepped flat areas, where water can percolate into the soil behind terrace steps and remain in the lower soil horizons, even if the terrace surfaces dry out. Notably, the high resistivity at 0.33 m in Sumedang (152–400 Ω·m) and Subang (~52–202 Ω·m) suggests that both had relatively dry topsoil at the time of survey (likely due to surface evaporation in the dry season). Meanwhile, Bandung’s shallow layer resistivity (8–20 Ω·m) was much lower – an unexpected result that points to localized moisture, perhaps from recent rainfall or irrigation, remaining in pockets of the upper Bandung soil. Below 0.5 m depth, however, Bandung’s resistivity climbed (50–238 Ω·m at 0.75–1.5 m), indicating that deeper layers were comparatively dry (or less saline). These EMI-derived depth profiles reinforce the stratification of moisture: Bandung had a wet shallow layer over dry subsoil, whereas Subang and Sumedang had drier shallow layers over wet subsoil.

- **Topographic and Textural Controls**

The contrasting moisture distributions observed can be explained by considering each site’s topography and soil composition. In steep hilly terrain (Bandung), rainfall tends to run off quickly and concentrate in lower areas, so upper slopes dry out fast. Bandung’s higher resistivity range and patchy moisture reflect this: much of the hillslope soil was dry, with only limited wetter zones where water may have temporarily pooled downslope. This agrees with prior observations in similar upland settings – for instance, Widodo et al. (2025a) [30] noted extremely dry topsoil on a Bandung slope with moisture only in deeper pockets, a result of rapid drainage of rainwater from the surface. In contrast, flat or gently sloping terrain (Subang) promotes water retention. Subang showed a narrow resistivity range (mostly exceptionally low values), implying uniformly high moisture across the site. With minimal slope, rain infiltrates and remains in the soil, and a high-water table can sustain moisture near the surface. The relatively high and stable conductivity conditions observed in the study area are consistent with previous hydrogeophysical investigations showing that irrigated agricultural soils commonly maintain relatively homogeneous electrical responses due to sustained soil moisture and limited drainage variability across flat terrains [23–31]. Terraced highlands (Sumedang) present an intermediate case: the terracing reduces overall slope length, allowing water to infiltrate behind each terrace step. Our results show Sumedang has a broad resistivity range (from very low to high), reflecting a mix of dry and wet zones – dry on exposed terrace surfaces and wetter in the protected, lower layers of soil. This aligns with Widodo et al. (2025b) [31], who found that Sumedang’s terraced fields had dry topsoil at the terrace fronts but significant moisture at depth, retained by the underlying clayey layers. Soil texture and mineral composition play a fundamental role in controlling the moisture retention capacity and electrical conductivity of soils. The proportion of clay, silt, and sand determines pore size distribution, permeability, and water-holding capacity, which in turn influence the electrical properties measured by geophysical methods. Fine-textured soils such as clay and loam typically have smaller pore spaces and higher specific surface areas, enabling them to keep larger amounts of water and dissolved ions compared to coarse-textured sandy soils. As a result, clay-rich soils generally exhibit lower electrical resistivity when saturated because the retained pore water facilitates electrical current flow through both electrolytic conduction and surface conduction mechanisms.

This relationship is clearly reflected in the soil profiles seen in the study areas. In Subang, the soil profile is dominated by clay and clay-loam materials derived from alluvial and coastal depositional environments. These fine-grained sediments show high water retention capacity and can store both moisture and dissolved salts within the soil matrix. Consequently, the resistivity values measured in Subang are consistently low throughout the subsurface profile, showing persistent soil moisture and relatively conductive conditions from the near surface to deeper layers.

In contrast, the Bandung site is characterized by a more heterogeneous soil profile containing significant amounts of sand derived from volcanic parent material. Sandy soil typically has larger pore spaces and lower capillary forces, resulting in reduced water retention and faster drainage. Field observations indicate that the sandy subsoil layers below approximately 30 cm depth in Bandung allow rapid infiltration and downward percolation of water, leaving the upper layers relatively dry after rainfall events. Once the limited moisture stored in the sandy layers dissipates through evaporation or drainage, the electrical resistivity increases significantly due to reduced ionic conductivity in the pore spaces.

The soil profile in Sumedang exhibits intermediate characteristics between the Subang and Bandung environments. The upper soil layers consist primarily of sandy or sandy-loam materials associated with volcanic deposits, while the deeper subsurface has clay-rich layers that originated from weathered volcanic ash and alluvial sediments. Integrated EMI and ERT analyses in this study found distinct zones corresponding to sandy soil, alluvial deposits, and clay-rich layers. The clay-rich horizons in the deeper subsurface function as moisture storage zones because of their high-water retention capacity and low permeability. This explains the occurrence of localized low-resistivity anomalies at depth, which correspond to higher soil moisture content. Meanwhile, the sandy-loam surface layers dry relatively quickly following rainfall or irrigation, producing higher resistivity values in the shallow subsurface.

Overall, the observed resistivity patterns strongly reflect the influence of soil texture on moisture distribution and electrical conductivity. Clay-dominated soils, such as those found in Subang and parts of Sumedang, tend to keep higher moisture content and show more conductive electrical behavior when water is present. In contrast, sandy soils, such as those observed in Bandung and the upper layers of Sumedang, display higher resistivity due to their lower water retention capacity and higher permeability. These findings are consistent with fundamental principles of soil physics and hydrogeophysics, which show that finer-grained soils not only keep greater amounts of water but also show higher exchange capacity and surface conductivity, both of which contribute to increased electrical conductivity under moist conditions.

- **Comparison of ERT and EMI, and Implications for Practice**

By combining ERT and EMI methods, we achieved a more nuanced understanding of the soil water distribution across varying scales. The ERT provided detailed vertical resistivity sections, confirming, for example, that Subang’s moisture extends from the surface downward and that Bandung’s subsurface is predominantly dry except for minor pockets. EMI, on the other hand, yielded horizontal slices of apparent resistivity that helped map how moisture varies spatially at fixed depths. The correspondence between the two methods is generally good – areas identified as conductive (wet) in the EMI maps align with low-resistivity zones in the ERT sections, and vice versa. This complementarity is in line with findings by Widodo (2024) [29], who reported that converting EMI data to resistivity allowed direct overlay with ERT results, revealing the same wet vs. dry zones in a Bandung-area field. Other researchers have also found ERT and EMI to be highly synergistic: for instance, EMI could rapidly survey large tracts to delineate conductivity patterns, which could then be calibrated and interpreted with the aid of co-located ERT profiles. In our study, the EMI data helped find subtle lateral variations (such as slightly drier patches within Subang’s field, or wetter foot-slope areas in Bandung) that the sparse ERT lines might have missed, while the ERT data helped resolve the vertical structure that EMI alone cannot provide. Together, these methods give farmers and land managers a powerful tool to non-invasively map soil moisture and salinity. Knowledge of these spatial patterns can support precision agriculture interventions – for example, adjusting irrigation schemes to account for drier spots on hill crests, improving drainage in overly wet lowland plots, or amending soils (through gypsum or organic matter) in areas prone to salinity buildup. Importantly, our results underscore that topography and soil texture must be considered when interpreting geophysical data: the same apparent conductivity value can show very different moisture statuses in a clay soil versus a sandy soil. Thus, ground-truthing (via soil pits or moisture sensors) remains a vital complement to geophysics. In sum, the integrated geophysical approach adopted here provides a rapid, cost-effective means to characterize soil water content across diverse terrains, which is invaluable for developing site-specific agricultural management strategies in hilly uplands, coastal lowlands, and terraced highlands alike.

The findings of this comparative study are consistent with the broader literature on near-surface geophysics in agriculture. They add Indonesian case studies to the growing body of work showing that geophysical methods can resolve fine-scale soil variability that matters for crop management [24,27]. By emphasizing how terrain and soil properties modulate the geophysical signals, we respond to calls in precision agriculture research for more context-aware interpretations of sensor data [26–28]. The results from Subang, Bandung, and Sumedang can inform similar geophysical assessments in other tropical regions where heterogeneous landscapes require tailored approaches to water management. Finally, the applicability of combined ERT–EMI techniques for improving soil moisture monitoring and, ultimately, guiding agricultural decision-making in diverse environmental settings of Indonesia.

### 4.1. Study limitations

Although the integrated ERT and EMI approach provides valuable insights into soil moisture distribution, several limitations should be acknowledged. First, resistivity measurements are influenced by multiple factors including soil texture, salinity, temperature, and mineral composition. Therefore, interpretation must be supported by field observations and soil sampling. Second, measurements were conducted during the dry season, which may not fully represent seasonal variations in soil moisture dynamics. Future studies should incorporate time-lapse monitoring to capture temporal changes in soil moisture distribution across agricultural landscapes.

## 5. Conclusions

Based on the integrated results of the resistivity (ERT) surveys, electromagnetic induction (EMI) data, and ground-truth test pits, we draw several conclusions regarding soil water content distribution in the three contrasting terrains:

**Terrain Influence on Moisture Distribution:** The Subang area (coastal lowland) exhibits a narrower range of resistivity values compared to Bandung and Sumedang, with consistently low resistivity indicating pervasive moisture and salinity. This is attributed to Subang’s relatively flat topography (low slope), which facilitates water retention and accumulation across the site. In the hilly Bandung area, the resistivity values span a much wider range, reflecting very dry conditions in upslope areas and occasional wetter zones downslope. The steep slopes in Bandung cause rainfall to rapidly drain to lower areas, resulting in predominantly dry soil on the hills and moisture concentration in limited zones downhill. Sumedang’s terraced slopes lead to intermediate behavior: its overall resistivity range is broad (due to dry exposed surfaces and wetter subsoils), and its high topographic gradient combined with terracing causes evaporation at terrace faces and moisture retention in interior soils.

**Soil Texture and Moisture/Salt Content:** The clay and loam soils in Subang (and the clayey topsoil in Bandung) can retain higher moisture and dissolved salts, leading to very low resistivity readings when wet. In Subang, the clay-loam profile was moist throughout, explaining its uniformly low resistivity. Bandung’s clay topsoil was extremely dry during the survey, resulting in high resistivity at the surface, but the fine sand layer beneath—while also relatively dry—exhibited some increased moisture at depth, indicated by localized low-resistivity pockets. Sumedang’s sandy soil naturally drains faster and holds less water, so it showed high resistivity when dry; however, where water was present (at depth or in less exposed zones), even the sandy soil produced low resistivity readings. Thus, clay-rich soils correspond to high conductivity (low resistivity) when moisture is present, whereas sandy soils require significant moisture to substantially reduce resistivity. In our observations, Subang had the highest overall moisture content (and likely salinity) evidenced by surface-reaching low resistivity, followed by Sumedang (moisture at depth), while Bandung had the lowest moisture and salinity overall.

**Integration of Geophysical Methods:** The combination of EMI and ERT proved effective for analyzing the soil water content in these agricultural lands when interpreted alongside test pit data. The apparent conductivity measured by EMI could be directly related to resistivity from ERT since one is essentially the opposite of the other, and we found no fundamental difficulties in linking the two datasets. In practice, converting EMI conductivity to resistivity allowed us to compare spatial patterns directly with the ERT sections, confirming that both methods identified similar wet and dry zones. The EMI provided rapid lateral coverage (maps of variability), while ERT provided detailed vertical sections, and together they offered a more comprehensive understanding than either would alone. This demonstrates that EMI and resistivity methods are complementary and can be jointly utilized for assessing soil moisture in farmlands, especially when supported by ground truth.

**Recommendations for Future Work:** For improved accuracy and to bolster the interpretations, future studies should include additional soil analyses, such as laboratory measurements of soil moisture content, salinity, and textural properties from samples taken at various depths. Measuring the soil’s mineral composition and organic content would help explain some of the geophysical observations (e.g., inherent soil conductivity unrelated to moisture). Such data would enhance the calibration of geophysical measurements to absolute moisture levels. Moreover, conducting surveys in different seasons (wet vs. dry) would be valuable to observe temporal changes in soil moisture distribution. These steps would further validate the geophysical approach and improve its utility as a tool for precision agriculture and sustainable land management in diverse terrains.

Ultimately, this work demonstrates that near-surface geophysical techniques can effectively delineate soil water content variability across different landscape types. Understanding the subsurface moisture distribution allows farmers and land managers to tailor irrigation and soil treatment strategies for each terrain. For example, the high moisture in Subang’s soil suggests a need for drainage management or cautious watering to avoid waterlogging, whereas the dry Bandung soils might require water conservation practices or soil amendments to improve moisture retention. The insights gained from ERT and EMI surveys, combined with direct soil observations, thus provide a scientific basis for recommending site-specific agricultural management practices to optimize crop growth and soil health.
