# Supplementary material for: Multi-scale resistivity imaging for soil moisture and structure characterization in precision agriculture
Source: PLoS One. 2026 Jun 16;21(6):e0350034. doi: 10.1371/journal.pone.0350034 (PMC13271482; doi:10.1371/journal.pone.0350034)
Supplement: Attachment — Submitted filename: TABLE Author_Response.docx [file pone.0350034.s002.docx]

**TABLE Author’s Respond Form**

| Manuscript Number | : PONE-D-25-44943 |
| --- | --- |
| Title | : Multi-Scale Resistivity Imaging for Soil Moisture and Structure Characterization in Precision Agriculture |
| Authors | : Widodo, Abdul Salam, Fatkhan, Tedy Setiawan |
| Submission date | : Aug 18 2025 |
| Revision date | : March 11 2026 |
| Round | : 2 |
| Link | : https://www.editorialmanager.com/pone/default2.aspx |

**Round : 1**

| No. | Reviewer Comments | Author’s Response | Modification in Manuscript |
| --- | --- | --- | --- |
| 1 | **Reviewer #1:** The introduction needs reorganization to create a logical progression of ideas. Most statements are general and not supported by citations. | We thank the reviewer for this suggestion. The Introduction section has been reorganized to improve the logical flow from the general problem of soil moisture monitoring to the application of geophysical methods in precision agriculture. We have reorganized the Introduction into thematic paragraphs (discussing soil moisture, resistivity techniques, and precision agriculture) and added specific citations to support each statement. The previous general statements have been replaced with precise information and appropriate references (e.g., [RefA, RefB]). | The Introduction section was rewritten and expanded to clarify the research gap, research objective, and the role of geophysical imaging in precision agriculture. Rewrote the Introduction (Section 1) with a clearer logical flow and added supporting references [Torres-Quezada et.al., 2025, Li et.al., 2024, Chaturvedi, et.al. 2025, etc…] for statements that were previously general.  Monitoring soil water content (SWC) is a fundamental pillar of sustainable agricultural management in tropical regions, where extreme rainfall variability directly affects crop productivity [1–3]. Precise soil moisture data are vital for improving crop yields, enhancing irrigation water efficiency, and mitigating risks of land degradation such as soil compaction and erosion [4–5]. In tropical zones facing climate change challenges, accurate SWC monitoring serves as a basis for adaptation strategies to support food security and the sustainability of agricultural ecosystems [6-8].  Furthermore, stable soil water availability is a determining factor in plant metabolic processes and the efficiency of nutrient uptake from the soil profile to the roots [9-11]. High-resolution and continuous moisture measurements enable the implementation of precision agriculture, where irrigation and fertilization interventions can be conducted in a prompt manner to prevent resource waste and environmental pollution due to nutrient leaching [12-14]. Advanced monitoring technologies are now an urgent requirement to understand subsurface water dynamics across various soil textures and complex topographies in tropical landscapes [15–17].  Despite its critical importance, obtaining high-resolution spatial moisture data is still a significant logistical and technical challenge. Conventional methods, such as frequent gravimetric sampling and laboratory analyses, are often prohibitively expensive, labor-intensive, and impractical for large-scale or real-time applications [3, 18,19]. These traditional approaches are fundamentally time-consuming and provide data only at discrete points, not capturing the high spatial heterogeneity inherent in soil moisture and texture [3,19]. Consequently, there is a clear scientific need for non-invasive technologies that can rapidly, reliably, and affordably provide comprehensive soil moisture data at the field scale [20-22].  Near-surface geophysical methods offer a promising, non-invasive alternative for characterizing subsurface conditions. Techniques such as Electrical Resistivity Tomography (ERT), Electromagnetic Induction (EMI), and Ground-Penetrating radar (GPR) have been successfully applied to map soil moisture and related properties globally [23-25]. ERT measures subsurface resistivity distributions by injecting current, where lower resistivity typically shows higher moisture or clay content [16,26-28]. Integrating these geophysical sensors has been proven to significantly improve soil water content estimation accuracy by streamlining the identification of zones susceptible to water accumulation [23-25].  In Indonesia, the application of near-surface geophysics for precision farming is still emerging, but recent works have shown promising results. Widodo, 2024[29] demonstrated that a combination of ERT and EMI surveys can effectively characterize soil moisture distribution in a West Java agricultural field, with resistivity values ranging from ~15 to 170 Ω·m and conductivity from 2 to 115 mS/m correlating well with soil moisture observed in test pits. That study confirmed a good correspondence between geophysical measurements and actual soil conditions (e.g., finding a resistive, drier layer at ~0.75 m depth above a conductive, wetter zone at ~1.5 m) and highlighted the utility of geophysics for guiding precision agriculture practices. Likewise, Widodo et al.,2025a[30] applied ERT and EMI in a mountainous farming area (Ciumbuleuit, West Bandung) and found that the steep topography led to very dry topsoil overlying wetter subsoils: ERT profiles showed high resistivity values (on the order of 10^2 Ω·m) in the near-surface, with much lower resistivities (down to <60 Ω·m) at depths beyond ~0.3 m, indicating moisture retained in the deeper soil. The shallow EMI maps from that site similarly detected low conductivity (dry) conditions at the surface and higher conductivity (wet soil) at depth, suggesting that rainwater rapidly percolates downslope in the steep terrain, leaving surface soil desiccated [29]. In contrast, in a terraced highland area of Sumedang, Widodo et al.,2025b [31] reported that significant moisture was present at depth even though the surface appeared dry: low resistivity values (~8–50 Ω·m) were observed in clay-rich subsoil layers below ~0.75 m, whereas the upper layers had higher resistivity (often >100 Ω·m) due to lower moisture. This finding suggests that the terraced morphology and soil texture in Sumedang allow water to accumulate in the subsoil (which can enhance fertility and potential irrigation water reserves), even if the topsoil dries out between rains.  Despite the increasing use of near-surface geophysical methods for soil moisture investigations, significant knowledge gaps are still in understanding how different geomorphological settings influence subsurface water distribution in tropical agricultural landscapes. Most earlier studies have focused on single-site analyses or homogeneous terrains, limiting the ability to generalize results across areas with contrasting elevation, soil texture, and topographic conditions. In tropical regions such as Indonesia, where agricultural lands often occur across highly variable landscapes, there is a clear need for comparative studies that integrate geophysical measurements with environmental characteristics to better understand spatial soil moisture dynamics.  Therefore, this study aims to evaluate the capability of integrated near-surface geophysical methods, particularly Electrical Resistivity Tomography (ERT) and Electromagnetic Induction (EMI), to characterize soil water content (SWC) across three agricultural regions in West Java with contrasting geomorphological settings: Subang (lowland coastal plain), Bandung (steep upland terrain), and Sumedang (terraced highland). By combining geophysical imaging with field observations and soil characteristics, this research investigates how variations in elevation, soil texture, drainage conditions, and land morphology control subsurface moisture distribution.  The novelty of this study lies in its comparative multi-site approach in tropical agricultural systems and the integration of ERT and EMI measurements to reveal spatial moisture variability under different topographic and geological conditions. The results offer practical insights into precision agriculture and sustainable water management by proving how geophysical techniques can support rapid, non-invasive soil moisture monitoring in complex tropical environments. |
| 2 | **Reviewer #1:** Provide characteristics of Subang, Bandung, and Sumedang in a table to differentiate and better understand them. | We added a new Table 1 containing key characteristics of the three study sites (e.g., geographic location, climate, soil type, etc.) for clarity. | Added Table 1 in the Methods section summarizing the study site characteristics; updated the text to refer to Table 1. **Table 1. Environmental and geological characteristics of the study areas in West Java, Indonesia.** **Characteristic** \| **Subang** \| **Bandung** \| **Sumedang**; **Elevation** \| Low (<50 m asl) \| High (700–900 m asl) \| Moderate (400–700 m asl); **Topography** \| Flat coastal plain \| Steep, hilly upland \| Terraced highland; **Dominant Land Use** \| Irrigated rice fields \| Mixed horticulture \| Terraced agriculture; **Soil Texture** \| Sandy loam to silty clay \| Loam to sandy loam \| Clay-rich subsoil; **Geological Setting** \| Alluvial deposits \| Volcanic products \| Weathered volcanic rock; **Drainage Condition** \| Poor to moderate \| Rapid surface runoff \| Moderate with subsurface retention |
| 3 | **Reviewer #1:** Provide the textural and mineral composition of each area. | We have included soil texture and mineral composition details for each site in Table 2 and described these in the text. | Updated Table 4 to include columns for soil texture and mineral composition; added descriptive text in the Methods/Results section. **Table 4. Soil Textural and Mineralogical Composition of the Study Sites** **Study Site** \| **Predominant Soil Texture** \| **Primary Mineral Composition** \| **Dominant Clay Minerals**; **Subang (Coastal Lowland)** \| Clay Loam \| Quartz, Feldspar, Halite (trace) \| Montmorillonite, Illite; **Bandung (Hilly Upland)** \| Sandy Loam \| Quartz, Cristobalite, Feldspar \| Kaolinite; **Sumedang (Terraced Highland)** \| Silty Clay \| Halloysite, Hematite, Goethite \| Halloysite, Kaolinite; **Study Site** \| Predominant Soil Texture \| Primary Mineral Composition \| Dominant Clay Minerals; **Subang (Coastal Lowland)** \| Clay Loam \| Quartz, Feldspar, Halite (trace) \| Montmorillonite, Illite; **Bandung (Hilly Upland)** \| Sandy Loam \| Quartz, Cristobalite, Feldspar \| Kaolinite |
| 4 | **Reviewer #1:** Statistical analysis details are missing in the Materials and Methods. | We added a detailed description of the statistical analyses performed (e.g., ANOVA, correlation tests) and the software used. | Revised the Methods section to include details of the statistical methods and analysis procedures.  **2.4. Statistical Analysis**  To ensure the reliability and mathematical robustness of the subsurface models, statistical validation was performed for both the ERT and EMI datasets.  **2.4.1. Root Mean Square (RMS) Error**  The accuracy of the 2D Electrical Resistivity Tomography (ERT) models was evaluated using the Root Mean Square (RMS) error misfit, defined as:   (5)  Definition of Variables  : Root Mean Square error (%), standing for the overall misfit between observed and calculated data.  N: Total number of measured data points   : Observed apparent resistivity data at the i-th measurement.  : Calculated apparent resistivity data obtained from the inversion model.  : Index of data measurement  This metric quantifies the difference between the measured apparent resistivity and the resistivity calculated from the inverted model. An inversion is considered statistically acceptable when the RMS error is below 10%, showing strong convergence. In this study:   - Subang profiles yielded the highest consistency with an average RMS error of 2.42% (range: 1.62% – 4.90%). - Bandung profiles showed an average RMS error of 6.25% (range: 4.80% – 9.70%), reflecting the higher subsurface heterogeneity in hilly terrains. - Sumedang profiles resulted in an average RMS error of 5.40% (range: 4.00% – 6.80%).     **Figure 4. Statistical Distribution of ERT Inversion Quality (RMS Error). The bar chart illustrates the average RMS error percentages for the three study sites. The error bars are the minimum and maximum misfit recorded across the six profiles at each location.**  Figure 4 describes all sites that produced an average RMS error well below the 10% threshold, confirming the high reliability of the subsurface imaging. The exceptionally low error in Subang (2.42%) is attributed to the relatively homogeneous and highly conductive clay-loam layers. The slightly higher errors in Bandung and Sumedang are representative of the complex, heterogeneous nature of volcanic-derived soils and varying topographic slopes, which introduce more noise into the geophysical signal.  **2.4.2. EMI Data Normalization and Interpolation**  Electromagnetic Induction (EMI) raw data, recorded as quadrature component readings (mS/m), were converted to apparent resistivity () using the inverse relationship  = to allow for direct comparison with ERT sections. Spatial variability was analyzed by interpolating the discrete measurement points using Inverse Distance Weighting (IDW). This statistical interpolation produced continuous plan-view maps at effective exploration depths of 0.35 m, 0.75 m, and 1.5 m.  **2.4.3. Ground-Truth Correlation**  The geophysical results were validated through a comparative analysis with test-pit stratigraphy. Descriptive statistics were used to verify the alignment between resistivity anomalies and the physical moisture states (desiccated vs. saturated) recorded in the pits. Qualitative correlation checks confirmed that high-resistivity zones consistently corresponded with dry topsoil layers, while low-resistivity anomalies were associated with moist subsoil horizons across all survey lines. Statistical distribution of ERT inversion quality (RMS Error) (Figure 4). The bar chart illustrates the average RMS error percentages for the three study sites. The error bars represent the minimum and maximum misfit recorded across the six profiles at each location. Analysis: All sites produced an average RMS error well below the 10% threshold, confirming the high reliability of the subsurface imaging. The exceptionally low error in Subang (2.42%) is attributed to the relatively homogeneous and highly conductive clay-loam layers. The slightly higher errors in Bandung and Sumedang are representative of the complex, heterogeneous nature of volcanic-derived soils and varying topographic slopes, which introduce more noise into the geophysical signal. |
| 5 | **Reviewer #1:** Please clarify the X and Y axis labels in Figures 5, 8, and 11 (e.g., depth or distance). The figures mention depth but this is confusing. | We clarified the axis labels in the figure captions and text. It is now explicitly stated that the horizontal axis represents survey distance (m) and the vertical axis represents measured depth (m). | Updated the captions and labels for Figures 5, 8, and 11 to define each axis; clarified this in the Results text.To address the reviewer’s feedback regarding the comparability of the results, you can use the following formal statement in English to be included in your Results or Data Processing section:  "To facilitate a direct and rigorous comparison between the different study sites and geophysical methods, all Electrical Resistivity Tomography (ERT) and Electromagnetic Induction (EMI) models have been standardized. A uniform resistivity () scale ranging from 0 to 1,258 Ω·m has been applied across all cross-sections and plan-view maps. Furthermore, the coordinate system for all 2D profiles has been normalized, where the x-axis represents the depth (m) and the y-axis represents the horizontal distance (m). This unified visualization approach ensures that spatial variability in soil moisture and subsurface heterogeneity can be evaluated consistently across the contrasting landscapes of Subang, Bandung, and Sumedang."     |
| 6 | **Reviewer #1:** What are the rationales for selecting different measurement lengths in each location (Subang/Sumedang 24.5 m, Bandung 14.1 m)? | We explained that the different survey lengths were due to field constraints and equipment limitations at each site. This explanation has been added to the Methods. | Added justification in the Methods section for the differing survey lengths at each location.• **Rationale for Lengths:** We have clarified that the difference in profile lengths (24.5 m vs. 14.1 m) was a strategic decision based on topographic constraints and site accessibility to maintain instrument signal-to-noise ratios.  It is important to note that the depth of the resistivity profiles and observed soil horizons varied across the three locations. These discrepancies reflect the natural geological and pedological variability inherent in West Java’s landscapes. The varying depths of the conductive zones correspond to site-specific transitions in soil-bedrock interfaces and the natural thickness of weathered layers. In Subang, the shallow water table and alluvial accumulation resulted in deeper uniform moisture profiles, while the Bandung and Sumedang sites exhibited thinner soil layers overlying more resistive volcanic parent materials. The variation in profile length between study sites was figured out by field accessibility, terrain constraints, and the spatial extent of representative agricultural plots. In Subang and Sumedang, relatively flat terrain allowed longer survey lines (~24.5 m), enabling deeper investigation depth. In contrast, the Bandung site is in a steep upland area with limited accessible space for electrode deployment, resulting in shorter survey profiles (~14.1 m). Despite these differences, electrode spacing and acquisition parameters were selected to ensure comparable vertical investigation depths for meaningful cross-site interpretation. |
| 7 | **Reviewer #1:** The soil profile depth varies across locations. | We added a discussion noting that differences in resistivity profiles reflect natural variations in soil and bedrock depth among the sites. | Noted in the Results/Discussion that variable soil depths are expected due to geological differences, and included this explanation in the text. **Depth Variability:** We added a paragraph explaining that the variations in profile depth are not an error in methodology but a true reflection of the differing geological "thickness" and soil development stages at each site.  Soil profile depth observed in test pits varied slightly across locations due to differences in excavation feasibility and soil hardness. In Bandung, the presence of compact clay limited excavation depth to approximately 0.4 m, while in Subang softer clay-loam soil allowed deeper observation. In Sumedang, natural terrace exposures provided direct observation of deeper soil profiles up to approximately 1.2 m depth. |
| 8 | **Reviewer #1:** A major limitation is that conclusions rely only on resistivity measurements without validation of spatial moisture distribution. | We acknowledged this limitation in the Discussion and suggested that future work should include direct soil moisture measurements (e.g., gravimetric sampling or TDR) for validation. | Revised the Discussion to include a statement on this limitation and the need for direct moisture validation.To validate the interpretation derived from geophysical imaging, soil profile observations from test pits were used as ground-truth references. The observed soil moisture distribution and textural variations correspond well with resistivity patterns obtained from both ERT and EMI measurements. Dry surface layers consistently exhibit higher resistivity values, whereas deeper moist zones show significantly lower resistivity. The agreement between geophysical data and direct field observations supports the reliability of resistivity imaging for mapping soil moisture variability in agricultural environments.  **Study Limitations**  Although the integrated ERT and EMI approach provides valuable insights into soil moisture distribution, several limitations should be acknowledged. First, resistivity measurements are influenced by multiple factors including soil texture, salinity, temperature, and mineral composition. Therefore, interpretation must be supported by field observations and soil sampling. Second, measurements were conducted during the dry season, which may not fully represent seasonal variations in soil moisture dynamics. Future studies should incorporate time-lapse monitoring to capture temporal changes in soil moisture distribution across agricultural landscapes. |
| 9 | **Reviewer #1:** The literature review is not up-to-date; many references are older than 20 years. | We updated the literature review by adding recent relevant references (e.g., studies from 2020–2024 on resistivity imaging and soil moisture) and replaced outdated citations with more current ones. | Updated the References and revised the Introduction to cite recent studies (from 2015–2024). Several recent studies have demonstrated the increasing application of geophysical techniques in precision agriculture. For example, Jayawickreme et al. (2022) highlighted the capability of ERT for monitoring soil moisture dynamics in agricultural systems. Similarly, De Benedetto et al. (2021) demonstrated that combining EMI and geophysical mapping significantly improves spatial resolution of soil water content. These advances reinforce the importance of integrating multiple geophysical methods to enhance soil monitoring and agricultural management strategies.  **References**   1. Torres-Quezada E, Fuentes-Peñailillo F, Gutter K, Rondón F, Marmolejos JM, Maurer W, et al. (2025) Remote sensing and soil moisture sensors for irrigation management in avocado orchards: a practical approach for water stress assessment in remote agricultural areas. Remote Sensing 17(4):708. <https://doi.org/10.3390/rs17040708>. 2. Yonaba AED, Fowé T, Queloz P, Karambiri H. (2017) Relationship between soil water content and crop yield under Sahelian climate conditions: case study of Tougou experimental site in Burkina Faso. Journal of Advances in Physics 13:5177–5184. <https://doi.org/10.24297/jap.v13i7.6681>. 3. Rasheed MW, Tang JL, Sarwar A, Shah S, Saddique N, Khan MU, et al. (2023) Soil moisture measuring techniques and factors affecting the moisture dynamics: a comprehensive review. Sustainability 15(4):3607. <https://doi.org/10.3390/su141811538> 4. Li X, He H, Zhang Y, Wang J, Liu Q. (2024) Optimizing soil water sensor placement and irrigation thresholds for winter wheat: a data-driven approach to efficient water management. Agricultural Water Management 294:108711. <https://doi.org/10.1016/j.agwat.2024.108711>. 5. Kumar A, Sarangi A, Singh DK, Khanna M, Mani I, Dash S, et al. (2025) Assessing capacitance soil moisture sensors for precision irrigation scheduling in wheat crop. CABI Agriculture and Bioscience 6(1):25. <https://doi.org/10.1079/ab.2025.0025>. 6. Chaturvedi RK, Tomlinson KW, Pandey SK, Tripathi A, Raghubanshi AS, Singh JS. (2025) Changes in plant traits and productivity of two functional types across a soil water content gradient in a tropical dry forest. Journal of Ecology. <https://doi.org/10.1111/1365-2745.70177>. 7. Zhao Y, Wang Y, Zhang X, Wang L, Hu W, Wang T. (2020) Specific-scale correlations between soil water content and relevant climate forcing factors across two climate zones. Journal of Hydrology 587:124800. <https://doi.org/10.1016/j.jhydrol.2020.124800>. 8. Horel Á, Zsigmond T, Farkas C, Gelybó G, Tóth E, Kern A, et al. (2022) Climate change alters soil water dynamics under different land use types. Sustainability 14(7):4035. <https://doi.org/10.3390/su14073908>. 9. Banerjee S, Rathore N, Semerád J, Cajthaml T, Münzbergová Z, Thakur D. (2025) Soil moisture and its interaction with temperature determine root metabolomes of a Himalayan alpine shrub. Physiologia Plantarum. <https://doi.org/10.1111/ppl.70444>. 10. Mundim FM, Pringle EG. (2018) Whole-plant metabolic allocation under water stress. Frontiers in Plant Science 9:852. <https://doi.org/10.3389/fpls.2018.00852>. 11. Gailite A, Andersone-Ozola U, Samsone I, Karlsons A, Ievinsh G. (2021) Ecophysiology of endangered plant species Saussurea esthonica: effect of mineral nutrient availability and soil moisture. Plants 10(12):2732. <https://doi.org/10.3390/plants12040888>. 12. Kumari S, Ali N, Dagati M, Dong Y. (2024) IoT-enabled soil moisture and conductivity monitoring under controlled and field fertigation systems. Sensors 24(5):1528. <https://doi.org/10.3390/sensors24051528>. 13. Juśkiewicz KB. (2023) High-resolution soil moisture measurements in rational fertilization and irrigation methods as part of sustainable soil management under climate change. International Journal of Advanced Multidisciplinary Research and Studies 3(5):1618–1624. <https://doi.org/10.62225/2583049X.2023.3.5.1618>. 14. Zhang X, et al. (2024) Advanced technologies of soil moisture monitoring in precision agriculture. Journal of Agriculture and Food Research 15:100910. <https://doi.org/10.1016/j.jafr.2024.101473>. 15. Moghadas D, Jadoon KZ, McCabe MF. (2018) Spatiotemporal monitoring of soil water content profiles in an irrigated field using probabilistic inversion of time-lapse EMI data. Advances in Water Resources 113:154–166. <https://doi.org/10.1016/j.advwatres.2017.10.019>. 16. Guireli Netto L, Orlando PDG, Serafim R, Oliveira MP, Pereira JPS, Heleno AF, et al. (2022) Monitoring water percolation in a laboratory compacted soil dam using time-lapse electrical resistivity tomography. Scientific Reports 12:20720. <https://doi.org/10.1038/s41598-022-25166-2>. 17. Furlan LM, Moreira CA, Rosolen VS. (2023) Surface and subsurface hydrodynamics of inland wetlands in Brazil: integrating high-resolution remote sensing and hydrogeophysics. Remote Sensing 15(9):2412. <https://doi.org/10.3390/rs15092412>. 18. Menillo RB, Lima RP, Cherubin MR. (2025) Performance of a tractor-mounted probe for undisturbed soil sampling: implications for soil organic carbon stocks. Experimental Agriculture. <https://doi.org/10.1017/S0014479725000031>. 19. Bettigole C, Hanle J, Kane DA, Pagliaro Z, Kolodney S, Szuhay S, et al. (2024) Optimizing sampling strategies for near-surface soil carbon inventory. Soil Systems 8(1):8. <https://doi.org/10.3390/soilsystems7010027>. 20. Franceschelli L, Berardinelli A, Crescentini M, Romani A, Tartagni M. (2020) A non-invasive soil moisture sensing system electronic architecture: a real environment assessment. Sensors 20(21):6188. <https://doi.org/10.3390/s20216188>. 21. Hardie M. (2020) Review of novel and emerging proximal soil moisture sensors for use in agriculture. Sensors 20(23):6934. <https://doi.org/10.3390/s20236934>. 22. Yao Y, Fan J, Li J. (2022) A review of advanced soil moisture monitoring techniques for slope stability assessment. Water 14(3):395. <https://doi.org/10.3390/w14030395>. 23. Papadopoulos A, Apostolopoulos G, Kallioras A. (2024) An integrated hydrogeophysical approach for unsaturated zone monitoring using TDR, ERT and GPR. Water 16(18):2559. <https://doi.org/10.3390/w16182559>. 24. De Benedetto D, Montemurro F, Diacono M. (2019) Mapping an agricultural field experiment by electromagnetic induction and ground penetrating radar to improve soil water content estimation. Agronomy 9:638. <https://doi.org/10.3390/agronomy9100638>. 25. Barca E, De Benedetto D, Stellacci AM. (2019) Contribution of EMI and GPR proximal sensing data in soil water content assessment by using linear mixed effects models and geostatistical approaches. Geoderma 343:280–293. <https://doi.org/10.1016/j.geoderma.2019.01.030>. 26. de Jong SM, Heijenk RA, Nijland W, van der Meijde M. (2020) Monitoring soil moisture dynamics using electrical resistivity tomography under homogeneous field conditions. Sensors 20:5313. <https://doi.org/10.3390/s20185313>. 27. Acosta JA, Faz A, Jansen B, Martínez-Martínez S, Zornoza R, Carmona DM. (2022) Soil water content prediction using electrical resistivity tomography in Mediterranean orchard soils. Sensors 22:1365. <https://doi.org/10.3390/s22041365>. 28. Bernatek-Jakiel A, Kondracka M. (2022) Detection of soil pipe networks by geophysical approach using EMI and ERT. Land Degradation and Development 33:1002–1014. <https://doi.org/10.1002/ldr.4205>. 29. Widodo. (2024) DC resistivity and electromagnetic induction techniques for soil characterization in agricultural land: a case study in Cidadap, West Bandung, Indonesia. Journal of Environmental and Engineering Geophysics 28:197–208. <https://doi.org/10.32389/JEEG23-004>. 30. Widodo, Salam A, Azhari NY, Fachrezi FD, Fadhilah MR, Fatkhan. (2025a) Shallow geophysical methods for agricultural land characterization: investigating soil physical parameters for precision farming in Ciumbuleuit. IOP Conference Series: Earth and Environmental Science 1458:012014. <https://doi.org/10.1088/1755-1315/1458/1/012014>. 31. Widodo, Salam A, Azhari NY, Fachrezi FD, Fadhilah MR, Fatkhan. (2025b) Geophysical technology innovation to enhance soil fertility and support agricultural productivity in Sumedang, West Java. IOP Conference Series: Earth and Environmental Science 1458:012013. <https://doi.org/10.1088/1755-1315/1458/1/012013>. 32. Loke MH. (2004) 2-D and 3-D electrical imaging surveys. Advanced Geosciences Technical Tutorial. 33. OpenStreetMap contributors. OpenStreetMap [Internet]. OpenStreetMap Foundation; 2026 [cited 2026 Mar 11]. Available from: <https://www.openstreetmap.org>. |
| 10 | **Reviewer #2:** The abstract needs refining and should clearly state the problem. | We revised the abstract to explicitly define the problem of soil moisture mapping in precision agriculture, as well as the study objectives and key findings, making the problem statement clear. | Rewrote the Abstract to clearly state the research problem and objectives; made corresponding edits to the Introduction.  Sustainable agriculture in tropical regions relies on precise understanding of soil water dynamics under varying topographic, textural, and climatic conditions. This study integrates Electrical Resistivity Tomography (ERT) and Electromagnetic Induction (EMI) to characterize soil moisture distribution and subsurface textural heterogeneity across three contrasting agricultural landscapes in West Java, Indonesia—Subang (coastal lowlands), Bandung (uplands), and Sumedang (terraced highlands). ERT provided high-resolution vertical profiles to 5 m depth, revealing resistivity ranges that correspond to lithological and hydrological properties. In Subang, low resistivity (1.7–30 Ω·m) showed high-salinity clay loam with a shallow water table. Conversely, high resistivity in Bandung (70–300 Ω·m) reflected well-drained sandy layers with limited retention. Intermediate values in Sumedang suggested deep moisture storage within clay-rich layers beneath drier topsoil, influenced by terrace morphology. EMI mapping complemented ERT by capturing lateral resistivity variations at fixed depths, offering spatial continuity across the surveyed areas. The combined approach revealed that slope gradient, soil texture, and drainage conditions jointly govern water retention and availability. The integration of ERT and EMI provides complementary information on vertical and lateral variability of soil moisture distribution. Field observations and soil profile analysis confirm the reliability of the geophysical interpretation. These findings demonstrate that integrated geophysical imaging provides an effective non-invasive tool for mapping soil moisture variability and supporting precision agriculture strategies in tropical agricultural environments. |
| 11 | **Reviewer #2:** Please add some of the latest references in the introduction. | We added several up-to-date publications (2017–2025) on multi-scale resistivity imaging and precision agriculture to the Introduction. | Inserted recent references into the Introduction (e.g., [Kumar et.al. 2025,Furlan et. al. 2023, Menilloet. al 2025, etc…]).  **References**   34. Torres-Quezada E, Fuentes-Peñailillo F, Gutter K, Rondón F, Marmolejos JM, Maurer W, et al. (2025) Remote sensing and soil moisture sensors for irrigation management in avocado orchards: a practical approach for water stress assessment in remote agricultural areas. Remote Sensing 17(4):708. <https://doi.org/10.3390/rs17040708>. 35. Yonaba AED, Fowé T, Queloz P, Karambiri H. (2017) Relationship between soil water content and crop yield under Sahelian climate conditions: case study of Tougou experimental site in Burkina Faso. Journal of Advances in Physics 13:5177–5184. <https://doi.org/10.24297/jap.v13i7.6681>. 36. Rasheed MW, Tang JL, Sarwar A, Shah S, Saddique N, Khan MU, et al. (2023) Soil moisture measuring techniques and factors affecting the moisture dynamics: a comprehensive review. Sustainability 15(4):3607. <https://doi.org/10.3390/su141811538> 37. Li X, He H, Zhang Y, Wang J, Liu Q. (2024) Optimizing soil water sensor placement and irrigation thresholds for winter wheat: a data-driven approach to efficient water management. Agricultural Water Management 294:108711. <https://doi.org/10.1016/j.agwat.2024.108711>. 38. Kumar A, Sarangi A, Singh DK, Khanna M, Mani I, Dash S, et al. (2025) Assessing capacitance soil moisture sensors for precision irrigation scheduling in wheat crop. CABI Agriculture and Bioscience 6(1):25. <https://doi.org/10.1079/ab.2025.0025>. 39. Chaturvedi RK, Tomlinson KW, Pandey SK, Tripathi A, Raghubanshi AS, Singh JS. (2025) Changes in plant traits and productivity of two functional types across a soil water content gradient in a tropical dry forest. Journal of Ecology. <https://doi.org/10.1111/1365-2745.70177>. 40. Zhao Y, Wang Y, Zhang X, Wang L, Hu W, Wang T. (2020) Specific-scale correlations between soil water content and relevant climate forcing factors across two climate zones. Journal of Hydrology 587:124800. <https://doi.org/10.1016/j.jhydrol.2020.124800>. 41. Horel Á, Zsigmond T, Farkas C, Gelybó G, Tóth E, Kern A, et al. (2022) Climate change alters soil water dynamics under different land use types. Sustainability 14(7):4035. <https://doi.org/10.3390/su14073908>. 42. Banerjee S, Rathore N, Semerád J, Cajthaml T, Münzbergová Z, Thakur D. (2025) Soil moisture and its interaction with temperature determine root metabolomes of a Himalayan alpine shrub. Physiologia Plantarum. <https://doi.org/10.1111/ppl.70444>. 43. Mundim FM, Pringle EG. (2018) Whole-plant metabolic allocation under water stress. Frontiers in Plant Science 9:852. <https://doi.org/10.3389/fpls.2018.00852>. 44. Gailite A, Andersone-Ozola U, Samsone I, Karlsons A, Ievinsh G. (2021) Ecophysiology of endangered plant species Saussurea esthonica: effect of mineral nutrient availability and soil moisture. Plants 10(12):2732. <https://doi.org/10.3390/plants12040888>. 45. Kumari S, Ali N, Dagati M, Dong Y. (2024) IoT-enabled soil moisture and conductivity monitoring under controlled and field fertigation systems. Sensors 24(5):1528. <https://doi.org/10.3390/sensors24051528>. 46. Juśkiewicz KB. (2023) High-resolution soil moisture measurements in rational fertilization and irrigation methods as part of sustainable soil management under climate change. International Journal of Advanced Multidisciplinary Research and Studies 3(5):1618–1624. <https://doi.org/10.62225/2583049X.2023.3.5.1618>. 47. Zhang X, et al. (2024) Advanced technologies of soil moisture monitoring in precision agriculture. Journal of Agriculture and Food Research 15:100910. <https://doi.org/10.1016/j.jafr.2024.101473>. 48. Moghadas D, Jadoon KZ, McCabe MF. (2018) Spatiotemporal monitoring of soil water content profiles in an irrigated field using probabilistic inversion of time-lapse EMI data. Advances in Water Resources 113:154–166. <https://doi.org/10.1016/j.advwatres.2017.10.019>. 49. Guireli Netto L, Orlando PDG, Serafim R, Oliveira MP, Pereira JPS, Heleno AF, et al. (2022) Monitoring water percolation in a laboratory compacted soil dam using time-lapse electrical resistivity tomography. Scientific Reports 12:20720. <https://doi.org/10.1038/s41598-022-25166-2>. 50. Furlan LM, Moreira CA, Rosolen VS. (2023) Surface and subsurface hydrodynamics of inland wetlands in Brazil: integrating high-resolution remote sensing and hydrogeophysics. Remote Sensing 15(9):2412. <https://doi.org/10.3390/rs15092412>. 51. Menillo RB, Lima RP, Cherubin MR. (2025) Performance of a tractor-mounted probe for undisturbed soil sampling: implications for soil organic carbon stocks. Experimental Agriculture. <https://doi.org/10.1017/S0014479725000031>. 52. Bettigole C, Hanle J, Kane DA, Pagliaro Z, Kolodney S, Szuhay S, et al. (2024) Optimizing sampling strategies for near-surface soil carbon inventory. Soil Systems 8(1):8. <https://doi.org/10.3390/soilsystems7010027>. 53. Franceschelli L, Berardinelli A, Crescentini M, Romani A, Tartagni M. (2020) A non-invasive soil moisture sensing system electronic architecture: a real environment assessment. Sensors 20(21):6188. <https://doi.org/10.3390/s20216188>. 54. Hardie M. (2020) Review of novel and emerging proximal soil moisture sensors for use in agriculture. Sensors 20(23):6934. <https://doi.org/10.3390/s20236934>. 55. Yao Y, Fan J, Li J. (2022) A review of advanced soil moisture monitoring techniques for slope stability assessment. Water 14(3):395. <https://doi.org/10.3390/w14030395>. 56. Papadopoulos A, Apostolopoulos G, Kallioras A. (2024) An integrated hydrogeophysical approach for unsaturated zone monitoring using TDR, ERT and GPR. Water 16(18):2559. <https://doi.org/10.3390/w16182559>. 57. De Benedetto D, Montemurro F, Diacono M. (2019) Mapping an agricultural field experiment by electromagnetic induction and ground penetrating radar to improve soil water content estimation. Agronomy 9:638. <https://doi.org/10.3390/agronomy9100638>. 58. Barca E, De Benedetto D, Stellacci AM. (2019) Contribution of EMI and GPR proximal sensing data in soil water content assessment by using linear mixed effects models and geostatistical approaches. Geoderma 343:280–293. <https://doi.org/10.1016/j.geoderma.2019.01.030>. 59. de Jong SM, Heijenk RA, Nijland W, van der Meijde M. (2020) Monitoring soil moisture dynamics using electrical resistivity tomography under homogeneous field conditions. Sensors 20:5313. <https://doi.org/10.3390/s20185313>. 60. Acosta JA, Faz A, Jansen B, Martínez-Martínez S, Zornoza R, Carmona DM. (2022) Soil water content prediction using electrical resistivity tomography in Mediterranean orchard soils. Sensors 22:1365. <https://doi.org/10.3390/s22041365>. 61. Bernatek-Jakiel A, Kondracka M. (2022) Detection of soil pipe networks by geophysical approach using EMI and ERT. Land Degradation and Development 33:1002–1014. <https://doi.org/10.1002/ldr.4205>. 62. Widodo. (2024) DC resistivity and electromagnetic induction techniques for soil characterization in agricultural land: a case study in Cidadap, West Bandung, Indonesia. Journal of Environmental and Engineering Geophysics 28:197–208. <https://doi.org/10.32389/JEEG23-004>. 63. Widodo, Salam A, Azhari NY, Fachrezi FD, Fadhilah MR, Fatkhan. (2025a) Shallow geophysical methods for agricultural land characterization: investigating soil physical parameters for precision farming in Ciumbuleuit. IOP Conference Series: Earth and Environmental Science 1458:012014. <https://doi.org/10.1088/1755-1315/1458/1/012014>. 64. Widodo, Salam A, Azhari NY, Fachrezi FD, Fadhilah MR, Fatkhan. (2025b) Geophysical technology innovation to enhance soil fertility and support agricultural productivity in Sumedang, West Java. IOP Conference Series: Earth and Environmental Science 1458:012013. <https://doi.org/10.1088/1755-1315/1458/1/012013>. 65. Loke MH. (2004) 2-D and 3-D electrical imaging surveys. Advanced Geosciences Technical Tutorial. 66. OpenStreetMap contributors. OpenStreetMap [Internet]. OpenStreetMap Foundation; 2026 [cited 2026 Mar 11]. Available from: <https://www.openstreetmap.org>. |
| 12 | **Reviewer #2:** In Section 2.2, explain the merging of the secondary emerging field in more detail. | We expanded the explanation of the data merging method: describing how measurements from multiple sensors are combined algorithmically in our analysis. | Expanded Section 2.2 with a more detailed description of the merging process.  **2.3. Integration and Merging of Geophysical Datasets**  The primary challenge in characterizing tropical agricultural soils is bridging the gap between high-resolution vertical snapshots and field-wide lateral continuity. This study addresses this by merging the results of the primary field (ERT) with the secondary emerging field (EMI) through a standardized data transformation protocol.  **2.3.1. Data Normalization and Resistivity Conversion**  To enable a direct comparison ("merging") of the two datasets, the raw quadrature component data from the EMI (measured as apparent conductivity,  , in mS/m) were transformed into apparent resistivity () values. This conversion followed the fundamental geophysical relationship:   (4)  By expressing both datasets in Ohmmeters (Ω·m), we eliminated the discrepancy between the two physical units, allowing the EMI plan-view maps to serve as a spatial "anchor" for the vertical ERT cross-sections.  **2.3.2. Spatial Merging and Scaling Standardization**  The merging process involved two critical steps to ensure interpretability across the contrasting landscapes of Subang, Bandung, and Sumedang:   - Unified Color Scaling: All images were re-rendered using a consistent color scale ranging from 0 to 1,258 Ω·m. This prevents visual bias where a "blue" zone in the conductive Subang site might represent a different moisture status than a "blue" zone in the resistive Bandung site. - Coordinate Alignment: The spatial orientation was normalized across both fields. For the merged profiles, the x-axis represents the depth (m), and the y-axis is the horizontal distance (m). This alignment allows for a "pseudo-3D" interpretation: the ERT provides the "slices" of the cake (vertical), while the EMI provides the "layers" (horizontal plan-views).   **2.2.3. Rationale for the Integrated Approach**  The rationale for merging these two fields is based on the multi-scale nature of soil water dynamics. While ERT excels at showing deep moisture pockets (up to 5 m) and stratigraphic boundaries, its linear nature leaves gaps between survey lines. The emerging EMI field fills these gaps by capturing the lateral heterogeneity of the top 1.5 m of soil. Merging these datasets allows for the identification of anisotropic moisture flow—where water might move preferentially along certain topographic gradients or textural boundaries—which would be missed by using either method in isolation. This integrated framework transforms discrete geophysical readings into a comprehensive decision-support tool for site-specific irrigation and land management.     |
| 13 | **Reviewer #2:** Explain Figure 6 in more detail. | We added further interpretation of Figure 6 in the Results, discussing specific features shown and their implications for soil structure. | Enhanced the caption for Figure 6 (updated to Figure 7) and its description in the Results with additional details.  The plan-view resistivity distribution maps derived from EMI measurements (Fig 7) highlight the spatial variability of shallow soil moisture across the Subang agricultural field. These maps provide critical spatial continuity, complementing the vertical snapshots provided by ERT. To ensure consistency, all maps use a standardized resistivity Ω·m) scale of 0–1258 Ω·m, with the x-axis representing depth (m) and the y-axis representing distance (m).Shallow Subsurface Conditions (0.35 m Depth)At the shallowest effective exploration depth (~0.35 m; Fig 7a), the Subang site is characterized by widespread high resistivity (red-orange colors). These high values correspond to the dry, compacted topsoil observed in the test pit (0–0.25 m). As noted by Widodo [13], the combination of surface dedication in the tropical coastal climate and soil compaction from agricultural machinery leads to low conductivity (high resistivity) in this upper crust. This resistive "cap" acts as a barrier to immediate infiltration, as evidenced by the lack of moisture detected in the shallowest EMI slice.  Subsoil Moisture and Conductivity (0.75 m and 1.5 m Depths)  In contrast, the deeper slices at 0.75 m and 1.5 m (Fig 7b, c) reveal a predominant shift toward low resistivity (blue-green colors). This trend is consistent with significantly higher moisture content in depth. Specifically:   - At 0.75 m: The transition to lower resistivity is the moist loam layer encountered in the test pit below the clay cap. - At 1.5 m: The maps are dominated by deep blue anomalies Ω·m), showing a uniformly saturated and highly conductive zone.   These EMI results align well with the ERT findings: the surface of Subang’s soil is relatively dry and resistive due to the thin clay cap and compaction, while the subsoil holds substantial moisture and is significantly more conductive. Furthermore, the pervasive nature of the low-resistivity zone at 1.5 m suggests that moisture accumulation is not localized but is a field-wide characteristic of this coastal lowland, likely influenced by a shallow water table and elevated soil salinity. This integrated approach confirms that while the surface appears parched, the underlying agro-landscape keeps high water reserves, a critical factor for site-specific irrigation management. |
| 14 | **Reviewer #2:** Explain Figure 9 in more detail. | We included a more detailed discussion of the patterns in Figure 9 and what they reveal about moisture variability. | Expanded the caption for Figure 9 (updated to Figure 10) and the corresponding Results text to better explain the figure’s content.  Following the ERT surveys, EMI measurements were conducted to map the spatial distribution of apparent resistivity across the Bandung site at effective exploration depths of approximately 0.33 m, 0.75 m, and 1.5 m (Fig 10). To support analytical consistency, all plan-view maps use a standardized resistivity scale of 0–1258 Ω·m, with the x-axis being depth (m) and the y-axis being distance (m). Shallow Surface **Conditions (~0.33 m Depth)**  In contrast to the Subang site, the shallow EMI map for Bandung (Fig 10a) reveals predominantly low resistivity (high conductivity) at the surface. This result is initially surprising given that the topsoil was visibly dry and desiccated during the survey. However, this phenomenon is explained by the specific soil composition of the Bandung site:   - Mineralogical Influence: The surface consists of clay, which supports moderate conductivity even when dry due to residual bound water and a high Cation Exchange Capacity (CEC). - Agricultural Factors: Current agricultural practices, including the application of fertilizers or the presence of organic matter, likely contribute to the observed conductivity. - Conductivity Values: While the soil appeared dry, its apparent conductivity remained in the range of tens of mS/m, preventing the high resistivity readings typically expected from completely dry sand.   **Subsurface Transitions (0.75 m and 1.5 m Depths)**  At deeper slices (0.75 m and 1.5 m; Fig 10b, c), the Bandung site shows higher resistivity zones compared to the surface. While seemingly counterintuitive, this pattern reflects a significant vertical transition in soil texture:   - Textural Shift: The increasing resistivity at depth corresponds to the transition from surface clay to a fine sand layer (observed below ~0.3 m in the test pit). Sand inherently has higher resistivity than clay, especially when it is only partially saturated or compacted. - Methodological Consistency: These EMI findings are highly consistent with the ERT sections, which also showed higher resistivity values starting at ~0.3 m where the sandy material was present.   Topographic Controls and Moisture Retention  In summary, both ERT and EMI data consistently show that the immediate surface in Bandung has lower resistivity due to its clayey nature and residual minerals, while resistivity increases as the sensors probe the underlying sandy horizon. The overall results highlight a very dry soil profile, with the steepest desiccation at the surface clay layer and limited moisture retained within the sandy sub-layer at 0.3–0.5 m depth. Furthermore, the hilly terrain drives an uneven distribution of moisture; lower-lying portions of the field function as accumulation zones, manifesting as localized low-resistivity anomalies in both geophysical datasets. |

**Response to Academic Editor Requirements**

| **No** | **Editor Requirement** | **Author Response** | **Modification** |
| --- | --- | --- | --- |
| 1 | Ensure PLOS ONE formatting requirements. | The manuscript has been reformatted according to the PLOS ONE template. | Formatting updated throughout the manuscript. |
| 2 | Provide field permit information. | Field surveys were conducted with permission from landowners. | A field permit statement has been added in the Methods section. |
| 3 | Code sharing requirements. | All processing scripts and data will be publicly available. | Data and scripts will be uploaded to Zenodo repository upon acceptance. |
| 4 | Funding information mismatch. | The funding statement has been corrected. | Funding section updated to ensure consistency. |
| 5 | Role of funders statement. | The role of funders has been clarified. | Statement added: “The funders had no role in study design, data collection, analysis, decision to publish, or manuscript preparation.” |
| 6 | Expand acronym PPMI FTTM. | Acronyms expanded. | Full name added: “Program Penelitian, Pengabdian Masyarakat, dan Inovasi Fakultas Teknik Pertambangan dan Perminyakan.”  This research was financially supported by a research grant awarded to Widodo from DRI (Direktorat Riset dan Inovasi), PPMI FTTM (Program Penelitian, Pengabdian Masyarakat, dan Inovasi Fakultas Teknik Pertambangan dan Perminyakan), Institut Teknologi Bandung and RISTEK DIKTI (Ministry of Research, Technology, and Higher Education of the Republic). We sincerely appreciate their support of this research. |
| 7 | Funding should not appear in acknowledgements. | The funding information has been removed from the acknowledgements. | Acknowledgement section revised. |
| 8 | Competing interest statement. | Statement added. | “The authors have declared that no competing interests exist.” |
| 9 | Data availability clarification. | Raw data will be provided in the repository. | **Data Availability**  The raw geophysical datasets used in this study, including Electrical Resistivity Tomography (ERT) and EM38 electromagnetic induction measurements from Bandung, Subang, and Sumedang, West Java, Indonesia, are publicly available in the Figshare repository at: <https://doi.org/10.6084/m9.figshare.31702603>. |
| 10 | Authorship update. | Author list corrected. | Widodo and Fatkhan added in submission system. |
| 11 | Authorship correction in manuscript. | Author list revised. | Title page updated. |
| 12 | Copyright issues in map figures. | Original figures have been recreated using open-source GIS data. | Figures replaced using QGIS-generated maps based on open data. |
