# Supplementary material for: Multi-scale resistivity imaging for soil moisture and structure characterization in precision agriculture
Source: PLoS One. 2026 Jun 16;21(6):e0350034. doi: 10.1371/journal.pone.0350034 (PMC13271482; doi:10.1371/journal.pone.0350034)
Supplement: Peer review history

# Peer review: Decision Letter 0

**DOI:** 10.1371/journal.pone.0350034.r001

**Authors:** Muammar Qadafi

19 Nov 2025

PONE-D-25-44943

Multi-Scale Resistivity Imaging for Soil Moisture and Structure Characterization in Precision Agriculture

PLOS ONE

Dear Dr. Widodo,

Thank you for submitting your manuscript to PLOS ONE. After careful consideration, we feel that it has merit but does not fully meet PLOS ONE’s publication criteria as it currently stands. Therefore, we invite you to submit a revised version of the manuscript that addresses the points raised during the review process.

Please submit your revised manuscript by Jan 03 2026 11:59PM. If you will need more time than this to complete your revisions, please reply to this message or contact the journal office at plosone@plos.org. When you're ready to submit your revision, log on to https://www.editorialmanager.com/pone/ and select the 'Submissions Needing Revision' folder to locate your manuscript file.

Please include the following items when submitting your revised manuscript:

- A rebuttal letter that responds to each point raised by the academic editor and reviewer(s). You should upload this letter as a separate file labeled 'Response to Reviewers'.
- A marked-up copy of your manuscript that highlights changes made to the original version. You should upload this as a separate file labeled 'Revised Manuscript with Track Changes'.
- An unmarked version of your revised paper without tracked changes. You should upload this as a separate file labeled 'Manuscript'.

If you would like to make changes to your financial disclosure, please include your updated statement in your cover letter. Guidelines for resubmitting your figure files are available below the reviewer comments at the end of this letter.

If applicable, we recommend that you deposit your laboratory protocols in protocols.io to enhance the reproducibility of your results. Protocols.io assigns your protocol its own identifier (DOI) so that it can be cited independently in the future. For instructions see: https://journals.plos.org/plosone/s/submission-guidelines#loc-laboratory-protocols. Additionally, PLOS ONE offers an option for publishing peer-reviewed Lab Protocol articles, which describe protocols hosted on protocols.io. Read more information on sharing protocols at https://plos.org/protocols?utm_medium=editorial-email&utm_source=authorletters&utm_campaign=protocols.

We look forward to receiving your revised manuscript.

Kind regards,

Muammar Qadafi

Academic Editor

PLOS ONE

Journal Requirements:

When submitting your revision, we need you to address these additional requirements.

1. Please ensure that your manuscript meets PLOS ONE's style requirements, including those for file naming. The PLOS ONE style templates can be found at

https://journals.plos.org/plosone/s/file?id=wjVg/PLOSOne_formatting_sample_main_body.pdf and https://journals.plos.org/plosone/s/file?id=ba62/PLOSOne_formatting_sample_title_authors_affiliations.pdf

2. In your Methods section, please provide additional information regarding the permits you obtained for the work. Please ensure you have included the full name of the authority that approved the field site access and, if no permits were required, a brief statement explaining why.

3. Please note that PLOS One has specific guidelines on code sharing for submissions in which author-generated code underpins the findings in the manuscript. In these cases, we expect all author-generated code to be made available without restrictions upon publication of the work. Please review our guidelines at https://journals.plos.org/plosone/s/materials-and-software-sharing#loc-sharing-code and ensure that your code is shared in a way that follows best practice and facilitates reproducibility and reuse.

4. We note that the grant information you provided in the ‘Funding Information’ and ‘Financial Disclosure’ sections do not match.

When you resubmit, please ensure that you provide the correct grant numbers for the awards you received for your study in the ‘Funding Information’ section.

5. Thank you for stating the following financial disclosure:

[DRI (Direktorat Riset dan Inovasi), PPMI FTTM, Institut Teknologi Bandung and RISTEK DIKTI (Ministry of Research, Technology, and Higher Education of the Republic)].

Please state what role the funders took in the study.  If the funders had no role, please state: "The funders had no role in study design, data collection and analysis, decision to publish, or preparation of the manuscript."

If this statement is not correct you must amend it as needed.

Please include this amended Role of Funder statement in your cover letter; we will change the online submission form on your behalf.

6. Please expand the acronym “PPMI FTTM” (as indicated in your financial disclosure) so that it states the name of your funders in full.

This information should be included in your cover letter; we will change the online submission form on your behalf.

7. Thank you for stating the following in the Acknowledgments Section of your manuscript:

[This research was financially supported by a research grant awarded to Widodo Widodo from DRI (Direktorat Riset dan Inovasi), PPMI FTTM, Institut Teknologi Bandung and RISTEK DIKTI (Ministry of Research, Technology, and Higher Education of the Republic). We sincerely appreciate their support of this research.]

We note that you have provided funding information that is currently declared in your Funding Statement. However, funding information should not appear in the Acknowledgments section or other areas of your manuscript. We will only publish funding information present in the Funding Statement section of the online submission form.

Please remove any funding-related text from the manuscript and let us know how you would like to update your Funding Statement. Currently, your Funding Statement reads as follows:

[DRI (Direktorat Riset dan Inovasi), PPMI FTTM, Institut Teknologi Bandung and RISTEK DIKTI (Ministry of Research, Technology, and Higher Education of the Republic)]

Please include your amended statements within your cover letter; we will change the online submission form on your behalf.

8. Thank you for stating the following in your Competing Interests section:

[NO authors have competing interests].

Please complete your Competing Interests on the online submission form to state any Competing Interests. If you have no competing interests, please state "The authors have declared that no competing interests exist.", as detailed online in our guide for authors at http://journals.plos.org/plosone/s/submit-now

This information should be included in your cover letter; we will change the online submission form on your behalf.

9. We note that your Data Availability Statement is currently as follows: [All relevant data are within the manuscript and its Supporting Information files.]

Please confirm at this time whether or not your submission contains all raw data required to replicate the results of your study. Authors must share the “minimal data set” for their submission. PLOS defines the minimal data set to consist of the data required to replicate all study findings reported in the article, as well as related metadata and methods (https://journals.plos.org/plosone/s/data-availability#loc-minimal-data-set-definition).

For example, authors should submit the following data:

- The values behind the means, standard deviations and other measures reported;

- The values used to build graphs;

- The points extracted from images for analysis.

Authors do not need to submit their entire data set if only a portion of the data was used in the reported study.

If your submission does not contain these data, please either upload them as Supporting Information files or deposit them to a stable, public repository and provide us with the relevant URLs, DOIs, or accession numbers. For a list of recommended repositories, please see https://journals.plos.org/plosone/s/recommended-repositories.

If there are ethical or legal restrictions on sharing a de-identified data set, please explain them in detail (e.g., data contain potentially sensitive information, data are owned by a third-party organization, etc.) and who has imposed them (e.g., an ethics committee). Please also provide contact information for a data access committee, ethics committee, or other institutional body to which data requests may be sent. If data are owned by a third party, please indicate how others may request data access.

10. Please amend the manuscript submission data (via Edit Submission) to include authors Widodo and Fatkhan.

11. Please amend your authorship list in your manuscript file to include authors Widodo Widodo and Fatkhan Fatkhan.

12. We note that Figures 3, 6, 9, 12, and 14 in your submission contain map and satellite images which may be copyrighted. All PLOS content is published under the Creative Commons Attribution License (CC BY 4.0), which means that the manuscript, images, and Supporting Information files will be freely available online, and any third party is permitted to access, download, copy, distribute, and use these materials in any way, even commercially, with proper attribution. For these reasons, we cannot publish previously copyrighted maps or satellite images created using proprietary data, such as Google software (Google Maps, Street View, and Earth). For more information, see our copyright guidelines: http://journals.plos.org/plosone/s/licenses-and-copyright.

We require you to either (1) present written permission from the copyright holder to publish these figures specifically under the CC BY 4.0 license, or (2) remove the figures from your submission:

1. You may seek permission from the original copyright holder of Figures 3, 6, 9, 12, and 14 to publish the content specifically under the CC BY 4.0 license.

We recommend that you contact the original copyright holder with the Content Permission Form (http://journals.plos.org/plosone/s/file?id=7c09/content-permission-form.pdf) and the following text:

“I request permission for the open-access journal PLOS ONE to publish XXX under the Creative Commons Attribution License (CCAL) CC BY 4.0 (http://creativecommons.org/licenses/by/4.0/). Please be aware that this license allows unrestricted use and distribution, even commercially, by third parties. Please reply and provide explicit written permission to publish XXX under a CC BY license and complete the attached form.”

Please upload the completed Content Permission Form or other proof of granted permissions as an "Other" file with your submission.

In the figure caption of the copyrighted figure, please include the following text: “Reprinted from [ref] under a CC BY license, with permission from [name of publisher], original copyright [original copyright year].”

2. If you are unable to obtain permission from the original copyright holder to publish these figures under the CC BY 4.0 license or if the copyright holder’s requirements are incompatible with the CC BY 4.0 license, please either i) remove the figure or ii) supply a replacement figure that complies with the CC BY 4.0 license. Please check copyright information on all replacement figures and update the figure caption with source information. If applicable, please specify in the figure caption text when a figure is similar but not identical to the original image and is therefore for illustrative purposes only.

The following resources for replacing copyrighted map figures may be helpful:

USGS National Map Viewer (public domain): http://viewer.nationalmap.gov/viewer/

The Gateway to Astronaut Photography of Earth (public domain): http://eol.jsc.nasa.gov/sseop/clickmap/

Maps at the CIA (public domain): https://www.cia.gov/library/publications/the-world-factbook/index.html and https://www.cia.gov/library/publications/cia-maps-publications/index.html

NASA Earth Observatory (public domain): http://earthobservatory.nasa.gov/

Landsat: http://landsat.visibleearth.nasa.gov/

USGS EROS (Earth Resources Observatory and Science (EROS) Center) (public domain): http://eros.usgs.gov/#

Natural Earth (public domain): http://www.naturalearthdata.com/

If the reviewer comments include a recommendation to cite specific previously published works, please review and evaluate these publications to determine whether they are relevant and should be cited. There is no requirement to cite these works unless the editor has indicated otherwise.

[Note: HTML markup is below. Please do not edit.]

Reviewers' comments:

Reviewer's Responses to Questions

**Comments to the Author**

1. Is the manuscript technically sound, and do the data support the conclusions?

The manuscript must describe a technically sound piece of scientific research with data that supports the conclusions. Experiments must have been conducted rigorously, with appropriate controls, replication, and sample sizes. The conclusions must be drawn appropriately based on the data presented.

Reviewer #1: Yes

Reviewer #2: Yes

**********

2. Has the statistical analysis been performed appropriately and rigorously?

Reviewer #1: No

Reviewer #2: Yes

**********

3. Have the authors made all data underlying the findings in their manuscript fully available?

The PLOS Data policy requires authors to make all data underlying the findings described in their manuscript fully available without restriction, with rare exception (please refer to the Data Availability Statement in the manuscript PDF file). The data should be provided as part of the manuscript or its supporting information, or deposited to a public repository. For example, in addition to summary statistics, the data points behind means, medians and variance measures should be available. If there are restrictions on publicly sharing data—e.g. participant privacy or use of data from a third party—those must be specified.

Reviewer #1: Yes

Reviewer #2: Yes

**********

4. Is the manuscript presented in an intelligible fashion and written in standard English?

PLOS ONE does not copyedit accepted manuscripts, so the language in submitted articles must be clear, correct, and unambiguous. Any typographical or grammatical errors should be corrected at revision, so please note any specific errors here.

Reviewer #1: Yes

Reviewer #2: Yes

**********

5. Review Comments to the Author

Please use the space provided to explain your answers to the questions above. You may also include additional comments for the author, including concerns about dual publication, research ethics, or publication ethics. (Please upload your review as an attachment if it exceeds 20,000 characters)

Reviewer #1: After careful observation of the manuscript, the following points are my specific comments

The introduction needs reorganization to create a logical progression of ideas. Most of the statements are general and not supported by citations.

Provide Subang, Bandung, and Sumedang characteristics in tabular form to differentiate and better understand.

Provide the textural and mineral composition of each area

Statistical analysis details are missing in the material and methods

Please clarify the x and Y axis labels in figures 5, 8, and 11. Is it a depth or length, for example, Bandung site 14.1 m length of the landscape or depth? In figures, it was mentioned as depth, it is supposed to be 0.4 m

What are the rationales in selecting different lengths of measurement in all three locations: Subang area-24.5 m, Sumedang area-24.5m, and Bandung area- 14.1m

Soil profile depth also varies across locations.

The major limitation of the study, conclusion relied only on resistivity measurements, ERT, EMI, which should be validated by the spatial moisture distribution, vertical, and horizontal measurements.

No exhaustive and up-to-date Literature collection in support of the present research; many of the references are older than 20 years.

Overall, the research topic may have merit, but the manuscript suffers in in-depth statistical analysis and comparison between selected areas. significant revision and professional editing are needed before it can be considered for publication.

Reviewer #2: It is a good work I am inclined to accept it after minor revision. Kindly refine the abstarct and write problem satatement clearly in abstarct. Kindly add some latesest reference in introduction.in section 2.2 kindly explain mergence of secondary emerging field in more deatil. kindly explain Fig.6 in more detail.kindly explain Fig. 9 in more detail.

**********

6. PLOS authors have the option to publish the peer review history of their article (what does this mean?). If published, this will include your full peer review and any attached files.

If you choose “no”, your identity will remain anonymous but your review may still be made public.

**Do you want your identity to be public for this peer review?**  For information about this choice, including consent withdrawal, please see our Privacy Policy.

Reviewer #1: **Yes:**  M.R. Umesh

Reviewer #2: No

**********

[NOTE: If reviewer comments were submitted as an attachment file, they will be attached to this email and accessible via the submission site. Please log into your account, locate the manuscript record, and check for the action link "View Attachments". If this link does not appear, there are no attachment files.]

To ensure your figures meet our technical requirements, please review our figure guidelines: https://journals.plos.org/plosone/s/figures

You may also use PLOS’s free figure tool, NAAS, to help you prepare publication quality figures: https://journals.plos.org/plosone/s/figures#loc-tools-for-figure-preparation.

NAAS will assess whether your figures meet our technical requirements by comparing each figure against our figure specifications.

# Author comment: Author response to Decision Letter 1

**DOI:** 10.1371/journal.pone.0350034.r002

13 Mar 2026

Dear Editor and Reviewers,

We would like to sincerely thank the Academic Editor and the reviewers for their careful evaluation of our manuscript and for providing constructive comments that have significantly improved the quality of this work. We have carefully revised the manuscript according to all suggestions and comments provided during the review process.

In response to the reviewers’ comments, several substantial improvements have been made to the manuscript. First, the Introduction section has been reorganized and expanded to provide a clearer scientific background, a stronger literature review, and a more explicit research gap regarding soil moisture monitoring in tropical agricultural environments. Several recent references have been incorporated to strengthen the context of the study.

Second, additional information describing the characteristics of the three study areas (Subang, Bandung, and Sumedang) has been included. A new comparative table has been added to the manuscript to summarize key environmental and soil characteristics of each location, which improves the clarity of the comparison between the different agricultural landscapes.

Third, to address the reviewer’s concern regarding statistical rigor, a new subsection describing the statistical analysis of resistivity variability has been added to the Materials and Methods section. Descriptive statistical parameters such as mean resistivity, standard deviation, and coefficient of variation are now used to quantify spatial variability among the study sites.

Fourth, several figure captions and axis descriptions have been clarified to avoid ambiguity in the interpretation of resistivity sections. The horizontal and vertical axes in all resistivity profiles now clearly indicate profile distance and depth, respectively.

Fifth, additional discussion has been added to clarify the rationale for different measurement lengths among the study sites. These variations are related to field accessibility and terrain conditions while maintaining comparable investigation depths.

Furthermore, the Discussion section has been substantially expanded to include validation of geophysical interpretations using soil profile observations and field data. A dedicated subsection discussing the limitations of resistivity-based soil moisture interpretation has also been included.

In addition, the manuscript has been revised to comply with all journal requirements, including clarification of funding information, competing interests statement, data availability statement, and author information. Figures containing map elements have been reviewed and revised to ensure compliance with open-access licensing requirements.

We believe that these revisions have significantly improved the clarity, scientific rigor, and overall quality of the manuscript. We hope that the revised manuscript now meets the publication criteria of PLOS ONE.

We sincerely appreciate the time and effort invested by the editor and reviewers in evaluating our manuscript and providing valuable feedback.

Thank you for your consideration.

Sincerely,

Widodo Widodo

(on behalf of all co-authors)

Institut Teknologi Bandung

Attachment Submitted filename: TABLE Author_Response.docx

# Editor report: Decision Letter 1

**DOI:** 10.1371/journal.pone.0350034.r003

**Authors:** Muammar Qadafi

7 May 2026

Multi-Scale Resistivity Imaging for Soil Moisture and Structure Characterization in Precision Agriculture

PONE-D-25-44943R1

Dear Dr. Widodo,

We’re pleased to inform you that your manuscript has been judged scientifically suitable for publication and will be formally accepted for publication once it meets all outstanding technical requirements.

Within one week, you’ll receive an e-mail detailing the required amendments. When these have been addressed, you’ll receive a formal acceptance letter and your manuscript will be scheduled for publication.

An invoice will be generated when your article is formally accepted. Please note, if your institution has a publishing partnership with PLOS and your article meets the relevant criteria, all or part of your publication costs will be covered. Please make sure your user information is up-to-date by logging into Editorial Manager at Editorial Manager® and clicking the ‘Update My Information' link at the top of the page. For questions related to billing, please contact billing support.

If your institution or institutions have a press office, please notify them about your upcoming paper to help maximize its impact. If they’ll be preparing press materials, please inform our press team as soon as possible -- no later than 48 hours after receiving the formal acceptance. Your manuscript will remain under strict press embargo until 2 pm Eastern Time on the date of publication. For more information, please contact onepress@plos.org.

Kind regards,

Muammar Qadafi

Academic Editor

PLOS One

Additional Editor Comments (optional):

Reviewers' comments:

# Editor report: Acceptance letter

**DOI:** 10.1371/journal.pone.0350034.r004

**Authors:** Muammar Qadafi

PONE-D-25-44943R1

PLOS One

Dear Dr. Widodo,

I'm pleased to inform you that your manuscript has been deemed suitable for publication in PLOS One. Congratulations! Your manuscript is now being handed over to our production team.

At this stage, our production department will prepare your paper for publication. This includes ensuring the following:

* All references, tables, and figures are properly cited

* All relevant supporting information is included in the manuscript submission,

* There are no issues that prevent the paper from being properly typeset

You will receive further instructions from the production team, including instructions on how to review your proof when it is ready. Please keep in mind that we are working through a large volume of accepted articles, so please give us a few days to review your paper and let you know the next and final steps.

Lastly, if your institution or institutions have a press office, please let them know about your upcoming paper now to help maximize its impact. If they'll be preparing press materials, please inform our press team within the next 48 hours. Your manuscript will remain under strict press embargo until 2 pm Eastern Time on the date of publication. For more information, please contact onepress@plos.org.

You will receive an invoice from PLOS for your publication fee after your manuscript has reached the completed accept phase. If you receive an email requesting payment before acceptance or for any other service, this may be a phishing scheme. Learn how to identify phishing emails and protect your accounts at https://explore.plos.org/phishing.

If we can help with anything else, please email us at customercare@plos.org.

Thank you for submitting your work to PLOS ONE and supporting open access.

Kind regards,

PLOS ONE Editorial Office Staff

on behalf of

Dr. Muammar Qadafi

Academic Editor

PLOS One
